# Socioeconomic differences in working life expectancy: a scoping review

**DOI:** 10.1186/s12889-024-18229-y

**Published:** 2024-03-07

**Authors:** Svetlana Solovieva, Astrid de Wind, Karina Undem, Christian Dudel, Ingrid S. Mehlum, Swenne G. van den Heuvel, Suzan J. W. Robroek, Taina Leinonen

**Affiliations:** 1 Finnish Institute of Occupational Health, P.O. Box 40, Työterveyslaitos, Helsinki 00032 Finland; 2 Amsterdam UMC location University of Amsterdam, Public and Occupational Health, Amsterdam, The Netherlands; 3 Amsterdam Public Health research institute, Societal Participation and Health, Amsterdam, The Netherlands; 4 National Institute of Occupational Health, Oslo, Norway; 5 Max Planck Institute for Demographic Research, Rostock, Germany; 6 Federal Institute for Population Research, Wiesbaden, Germany; 7 Max Planck – University of Helsinki Center for Social Inequalities in Population Health, Rostock, Germany; 8 Max Planck – University of Helsinki Center for Social Inequalities in Population Health, Helsinki, Finland; 9 Institute of Health and Society, University of Oslo, Oslo, Norway; 10 Department of Occupational and Environmental Medicine, Bispebjerg and Frederiksberg Hospitals, Copenhagen, Denmark; 11 Department of Public Health, University of Copenhagen, Copenhagen, Denmark; 12 TNO – Netherlands organisation for applied scientific research, Leiden, the Netherlands; 13 Department of Public Health, Erasmus University Medical Center, Rotterdam, the Netherlands

**Keywords:** Education, Healthy working life expectancy, Income, Occupational class, Working years lost

## Abstract

**Background:**

In the last decade, interest in working life expectancy (WLE) and socioeconomic differences in WLE has grown considerably. However, a comprehensive overview of the socioeconomic differences in WLE is lacking. The aim of this review is to systematically map the research literature to improve the insight on differences in WLE and healthy WLE (HWLE) by education, occupational class and income while using different ways of measuring and estimating WLE and to define future research needs.

**Methods:**

A systematic search was carried out in Web of Science, PubMed and EMBASE and complemented by relevant publications derived through screening of reference lists of the identified publications and expert knowledge. Reports on differences in WLE or HWLE by education, occupational class or income, published until November 2022, were included. Information on socioeconomic differences in WLE and HWLE was synthesized in absolute and relative terms.

**Results:**

A total of 26 reports from 21 studies on educational and occupational class differences in WLE or HWLE were included. No reports on income differences were found. On average, WLE in persons with low education is 30% (men) and 27% (women) shorter than in those with high education. The corresponding numbers for occupational class difference were 21% (men) and 27% (women). Low-educated persons were expected to lose more working years due to unemployment and disability retirement than high-educated persons.

**Conclusions:**

The identified socioeconomic inequalities are highly relevant for policy makers and pose serious challenges for equitable pension policies. Many policy interventions aimed at increasing the length of working life follow a one-size-fits-all approach which does not take these inequalities into account. More research is needed on socioeconomic differences in HWLE and potential influences of income on working life duration.

**Supplementary Information:**

The online version contains supplementary material available at 10.1186/s12889-024-18229-y.

## Introduction

Despite increased longevity, the average length of working life remains relatively short as compared to life expectancy [1, 2]. According to Eurostat statistics, working life expectancy (WLE) at age 15 in Europe in 2021 was 38.2 years among men and 33.7 years among women, respectively [3], while life expectancy at this age was 62.6 years (men) and 68.3 (women). Earlier studies reported significant gender, educational and occupational class differences in WLE [1, 4–6].

WLE denotes the time that a person is expected to participate in working life after a given age [7]. The measure is similar to life expectancy but with permanent exit from working life as the final state, irrespective of how the labour market is left (e.g., retirement or death). WLE is a population summary measure, which is forecasting a duration of working life of all individuals in a particular study population based on cumulative labour market attachment. It does not determine how long an individual will actually work during the remaining lifespan.

Different terms are used in the literature for the WLE, e.g., labour force expectancy, labour market life expectancy, active life expectancy [1]. A principal conceptual distinction of these terms is in how participation in working life is defined.

Participation in working life can be defined in several ways. The broadest definition refers to being economically active and thereby available to the labour market, i.e., being in the labour force as either employed/self-employed or unemployed. WLE estimated based on labour force participation rates is sometimes called labour force expectancy or economic activity expectancy [1]. In a narrower definition, participating in working life is restricted to being employed and is sometimes referred to as employment life expectancy [8]. An even stricter definition of working life refers to productive work only, i.e., not being e.g., in sickness absence or in subsidized employment [9]. The latter definition of participation in working life takes into account possible temporary interruptions of work due to ill-health or other reasons (e.g. unemployment, studying or care activities) more comprehensively than the former two.

WLE takes into consideration the complex interplay between changes in life expectancy and age-specific patterns of labour market behaviour of individuals in the population, which cover entry patterns at a young age, exit schedules at old age, temporary exit and re-entering employment and productive work participation during the lifespan. It differs from the average duration of working life, calculated based on average ages at which individuals enter and exit from the labour market [10]. The time that a person at a given age is expected to spend in other labour market states than employment determines working years lost (WYL). The sum of WLE and WYL indicates the potential remaining working years after a specific age. The WYL can be decomposed by reason due to which working years were lost (e.g., unemployment, receiving disability benefits or retirement).

Poor health, chronic diseases, and reduced work ability were found to be associated with withdrawal from the labour force due to disability, early retirement and accidental death, especially among older adults [11–17]. In order to incorporate longevity, health status and labour force participation into one population metric, healthy working life expectancy (HWLE) was introduced [18, 19]. HWLE is defined as the time that a person at a given age is expected to be healthy and participate in working life until permanent withdrawal from the labour market. This indicator has been in use for several years utilizing varying definitions of “being healthy”, e.g., good self-rated health [20–23] or absence of disabilities [24].

Socioeconomic differences in labour market participation and age of withdrawal from paid employment are well established [25–27]. Workers with low socioeconomic position were more prone to earlier exit from the labour market even after controlling for ill-health [25]. Education, occupation and income are the three most common indicators of socioeconomic position. Even though they are correlated, they capture distinct aspects of socioeconomic position and thus are not interchangeable [28, 29]. Recent studies with a primary focus on socioeconomic differences reported a substantially lower WLE among persons with low education and among manual workers [5, 6, 30].

In the last 10 years, interest in WLE and socioeconomic differences in WLE has grown considerably, and an increasing number of papers are published each year. According to a recent narrative review on indicators and determinants of the years of working life lost, persons with low socioeconomic position have lower WLE and more years of working life lost than those with high socioeconomic position [16]. However, a comprehensive overview of the socioeconomic differences in WLE and HWLE is lacking. With this review, we aim to provide an overview of this quickly expanding body of research. Specifically, we aim to improve the insight on differences in WLE and HWLE by education, occupational class and income while using different ways of measuring and estimating WLE and define future research needs.

## Method

To review the existing reports on socioeconomic differences in WLE is demanding because of the vast diversity in fields of research and methodological approaches. We chose to conduct a scoping review instead of a systematic review because the former is better able to map the available research literature and answer broader questions [31, 32]. Furthermore, a scoping review allows to clarify the complex concept of WLE, incorporate various study designs and estimation approaches in both published and grey literature and identify knowledge gaps. In contrast, systematic reviews often have a narrow research question, such as the strength of evidence for association, effectiveness of treatments/interventions. To conduct the scoping review, we followed the five-step methodological framework proposed by Arksey and O’Malley [33].

### Step 1: identifying the research question(s)

We identified three research questions for the scoping review: (1) what knowledge is available on socioeconomic differences in WLE and HWLE, (2) do socioeconomic differences in WLE and HWLE vary across different operationalisations of WLE and HWLE (i.e. different ways of measuring and estimating WLE) and (3) what are the challenges that future research on WLE and HWLE should address?

### Step 2: identifying relevant studies

We identified relevant studies by searching published articles in the electronic databases Web of Science, PubMed and EMBASE until November 2022, using a combination of the following keywords in text: (“working life expectancy” or “work life expectancy” or “working life duration” or “working years lost” or “labour market affiliation” or “healthy working life expectancy”) and (socioeconomic or education or income or occupation or “occupational class” or “social class”). The search was complemented by additional relevant publications derived through search in Google Scholar, screening of reference lists of the identified publications. Additional references were included according to the knowledge of the authors. We limited our searches to reports written in English but did not use any year of publication limit.

### Step 3: study selection

We scanned titles and abstracts, applying three inclusion criteria: (1) the main report’s focus was on WLE or HWLE, (2) reports include a description of estimation method for WLE or HWLE and 3) reports presented results on socioeconomic factors associated with WLE or HWLE. Reports focusing on WLE or HWLE but not showing results on socioeconomic differences in these measures, as well as reports focusing on individual-level measures of working life duration were therefore excluded from the current review. To identify eligible articles, titles and abstracts were screened and full-text reading of potentially relevant articles was performed by the first author. When abstracts provided insufficient information to make a decision on exclusion or inclusion of the reports, a full text was reviewed. Decisions about ambiguous papers were taken together by the authors.

### Step 4: charting the data

Data extraction was performed by the first author using predefined tables. The headings in the table were checked and verified by all co-authors. In addition to bibliographic information (authors’ names, publication year, and study location), characteristics of the study population and data sources, we extracted key results and information on operationalization of socioeconomic position and WLE as well as the method and types of working life tables used to estimate WLE. We extracted key results and information on operationalization of socioeconomic factors and WLE (listed labour market states), as well as the method and types of working life tables used to estimate WLE. Two approaches have been used to estimate WLE or HWLE. The prevalence-based approach, sometimes called Sullivan’s method, is based on the prevalence of labour market states and mortality rates, while the incidence-based approach is based on incidence rates that capture transitions between states. Furthermore, the WLE can be estimated using either cohort or period life tables [5, 34, 35]. Period life tables can be constructed based on data from one or a few years. Essentially, the age-specific labour market conditions observed in this brief period are used to cover complete working lives, thus representing synthetic working trajectories. In contrast, cohort life tables are constructed based on real working life trajectories. Selection of the approaches is commonly driven by data availability. For those reports that in addition to WLE presented results for WYL we also extracted information on socioeconomic differences in WYL.

The data extraction was checked and verified by all co-authors and in more detail by TL and AdW.

We did not perform a quality assessment of the included studies as this review was aiming to map published empirical research in the field regardless of the quality of the studies.

### Step 5: collating, summarizing, and reporting

For quantitative data synthesis, we included only the studies with population-representative data. To synthesize information on the magnitude of educational, occupational class and income differences in WLE, HWLE and WYL, we calculated the absolute difference (years) in the outcome of interest between the highest and lowest socioeconomic categories. In order to examine variation in socioeconomic differences across different age groups and across different operationalizations of WLE, we also calculated the relative difference by dividing the years-difference between the highest and lowest socioeconomic categories by remaining potential working years. The remaining potential working years were calculated as years from the specific age (for which expectancy was estimated) until age 65. For example, for WLE at age 30, the remaining potential working years equalled 35 years.

## Results

### Literature search and exclusion of studies

**Fig. 1:**
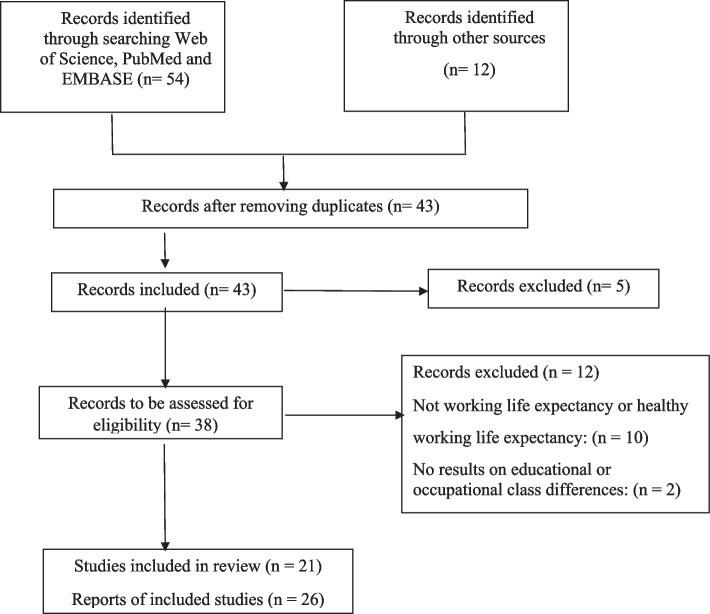
Flow chart of selection of sources of evidence

Figure 1 presents a flow chart of the literature search and the inclusion and exclusion of records. The literature search of reports published before November 2022 yielded 54 records, including 23 duplicates. In addition, 12 potentially relevant publications were selected from the reference lists or suggested by the experts. A total of 43 records were screened based on title and abstract. Of the 38 publications eligible for full-text reading, 26 reports from 21 studies were included into the review [1, 2, 4–6, 21, 24, 30, 34, 36–52]. Six of the twelve excluded reports were excluded because they focused on retirement age instead of WLE. Excluded reports and explanation for their exclusion based on the full-text reading are shown in the Supplement (Table S1). Of the duplicated reports from the same study the results of the first report were included into quantitative analyses (Supplementary Table S2).

### Overall study characteristics

Table 1 presents a description of the included reports. Studies were conducted in the United States (*n* = 7), Finland (*n* = 3), Spain (*n* = 3), The Netherlands (*n* = 3), United Kingdom (*n* = 3), Germany (*n* = 2), Italy (*n* = 1), Denmark (*n* = 1), and Turkey (*n* = 1). Two reports presented results for several European countries. All reports were published between 1990 and 2022, with the majority (16 out of 26) published during the past five years.

**Table 1:** Characteristic of the 26 included reports. Abbreviations explained in footnote

Author, year	Study sample and setting	Data source	Labore market states	Method of WLE estimation	Results presented
***Educational differences***					
Hayward & Lichter 1998 [37]^a^	45–59 years old men; *N* = 5 020; 1966–1983; U.S.	The National Longitudinal Survey of Older Men; Self-reports.	In the labour force, retirement, disability, died.	Increment–decrement model with transition rates derived from multivariate hazard models for each type of transition. Cohort data. No age censoring.	WLE at age 45 and 55 by education The estimates of time in other three states by education are also available.
Millimet et al. 2003 [38]^2^	17–86 years old; *N* = 200 916; 1992–2000; U.S.	Bereau of Labour Statistics and Current Population Surveys 1990–2000; Self-reports.	Employed, unemployed, inactive.	Increment–decrement model using relative frequencies (transitional probabilities calculated for entire sample) and econometric model (transitional probabilities calculated for subgroups). Period data. No age censoring.	WLE for the 21–70 age range by gender, education and initial status (employed, unemployment and inactivity).
Karlsson et al. 2009 [39]	0–90 years old; *N* = 9 865, 78% (*N* = 7 735) of the 26–60 years old; 1991–2004; U.K.	First fourteen waves of the British Household Panel Survey (BHPS); Self-reports.	Employment, health status, retirement, died.	Simulation of life trajectories based on estimated hazard rates for mortality, employment, and retirement, utilizing econometric approach. Period data.	Life expectancy (LE), healthy life expectancy (HLE) and WLE at age 50 by gender and combination of employment, education, health status at initial state.
Millimet et al. 2010 [40]^b^	17–71 years old; 1992–2001; U.S.	Bereau of Labour Statistics and Current Population Surveys 1990–2000; Self-reports.	Employed, unemployed, inactive.	Increment–decrement model using relative frequencies (transitional probabilities calculated for entire sample) and econometric model (transitional probabilities calculated for subgroups). Period data. No age censoring.	WLE for the 30–60 age range by gender, initial status (employed, unemployment and inactivity), education, race, marital status
Skoog et al. 2011 [41]^3^	16–75 + years old; *N* = 471 722; 2005–2009; U.S.	Current Population Survey; Self-reports.	Economically active, inactive, dead.	Markov increment-decrement worklife expectancy model. Period data. Censoring at age of 76.	WLE expectancy for the 16–75 age range conditional on initial state (active/ inactive) by gender and education.
Nurminen 2012 [42]	15–74 years old; 2000–2010; Finland	Finnish Labour Force Survey; Self-reports.	Employed, unemployed, economically inactive, dead.	Multistate life table method. data. Censoring at age of 65.	Partial WLE, unemployment and inactivity expectancy for the 15–60 age range (5-year interval) by gender and education.
Krueger & Slesnick 2014 [43]^c^	18–70 years old non-institutional population; *N* = 635 555; 2009–2013; U.S.	Current Population Survey (CPS); Self-reports.	In the labour force (active, in market work), active, in non-market work, inactive, died.	Markov increment-decrement worklife expectancy model. Period data. Censoring at age of 71.	WLE (years active in the market) for the 25–70 age range, conditional on initial state (active in the labour market work/active in non-market work) by gender and education.
Özer 2014 [44]	16 + years old women; *N* = 11 762; 2009–2010; Turkey	Income and Living Conditions Surveys, TURKSTAT; Self-reports.	Being active (employed or unemployed), being inactive (out of labor force).	Multistate working life tables based on increment–decrement model. Period data. Censoring at age of 75.	WLE for the 20–70 age range, by education, conditional on initial state (active or inactive). The expected time being inactive by education, conditional on initial state is also shown.
Loichinger & Weber 2016 [1]	15–74 years old; 28 EU countries; 1983–2013	Eurostat database based on European Labor Force Survey and the Human Mortality Database (based on registers); Self-reports.	Economically active, inactive.	Sullivan method, with prevalence rates aggregated by 5-year age-groups. Period data. Censoring at age of 75.	WLE (remaining active life expectancy) at age 50 in 2009 by country, gender and education for 11 EU countries.
Dudel & Myrskylä 2017 [34]^d^	50–99 years old; *N* = 30 254; 1992–2011; U.S.	The Health and Retirement Study (HRS); Self-reports.	Employed, retired (retired or 70 + years old and out of the labor force, out of the labor force (under 70 yrs. old and not working) or unemployed, died.	Multistate Markov model, annual transitions. WLE was weighted but not conditioned on the initial state Period data. No age censoring.	WLE at age 50 (years and %) by time period (1993–1997, 1998–2002, 2003–2007, and 2008–2011); and by gender/race/ education
Stanek & Requena 2019 [46]	50 + years old; *N* ~ 180 000 individuals; 2000–2014; Spain	An Economically Active Population Survey and life tables for the Spanish population (the Spanish National Statistics; Self-reports.	Employed, unemployed, retired, being economically inactive (other than retirement and unemployment).	Sullivan method. Period data. No age censoring.	WLE at age 50 by gender and education in 2012 The expected time in other three states by gender and education are also shown.
van der Noordt et al. 2019 [24]	55–65 years old; *N* = 1074; 1992–1996; 2002–2006; 2012–2016; The Netherlands	Three population-based cohorts; Self-reports.	In the workforce without disability, in the workforce with disability, out of the workforce.	Multistate Markov model based on annual transitions. Cohort data. Censoring at age of 68.	Total WLE, WLE with disability and WLE without disability at age 58 by education.
Robroek et al. 2020 [6]	16–66 years old; *N* = 4 999 947; 2001–2014; The Netherlands	Social statistical database (Statistics Netherlands); monthly information on the main income components, Mortality statistics. Registers.	Paid employment, disability benefits, unemployment, no income through paid employment, early retirement, being a student, emigration, died.	Multistate model biased on monthly transitions. Period data. Censored at age of 66.	WLE between ages 16–66, 30–66 and 50–66 by gender and education. The expected time in other three states by gender and education are also shown.
Lorenti et al. 2020 [47]^d^	50 + years old; *N* = 19 062; 2008–2014; U.S.	Health and Retirement Study (HRS) - a longitudinal survey; Self-reports.	Working, disabled, not working (includes inactive, unemployed, retired), died or age 100 years	Multistate Markov model based on annual transitions. Period data. No age censoring.	Working and disability LE at age 50 by gender, childhood disadvantage and education.
Weber & Loichinger 2020 [2]	50–69 years old; 2017; 26 European countries	Eurostat database based on the European Labour Force Survey (EU-LFS); Period life tables for ages 0–85 + by sex and country from Eurostat; Data from SHARE Survey wave 1 (2004) - wave 7 (2017); Self-reports.	Economically active, inactive.	Sullivan method. Period data. Censoring at age of 60 (for 50–59 years old group) and 70 (for 60–69 years old group).	The number of years a person is expected to be economically active and generally, physically, and cognitively healthy (called working, healthy, physical, and cognitive life expectancy, respectively) between age 50–59 and 60–69 by country and gender.
Nexø et al. 2021 [49]	18–65 years old; *N* = 115 118 (with diabetes) and *N* = 663 656 (without diabetes); 2000–2016; Denmark	Several linked Danish national registers.	Work, long-term sickness absence, temporary absence, unemployment, disability pension, died.	Multistate Cox proportional hazard model with weekly transitions between the states. Period data. Censoring at age of 65.	WLE for the 30–60 age range by gender, education and cohabitant status. The expected time in other states by gender, education and cohabitant status is also shown.
Tetzlaff et al. 2022 [52]	18–69 years old; *N* = 1 193 061; 2011–2013; Germany	The statutory health insurance provider (AOK Lower Saxony, AOKN); Registers.	In the labour force (employed and unemployed) and not in the labour force (other).	Markov increment-decrement worklife expectancy model using two states life table. Period data. Censoring at age of 70.	Years in the labour market at age 18 and 50 by gender and education
Schram et al. 2022 [30]	50–66 years old; *N* = 11 800; 2010, 2015; The Netherlands	The Dutch longitudinal Study on Transitions in Employment, Ability and Motivation (STREAM). Registers.	Years in paid employment, involuntary work exit (unemployment, disability benefits), voluntary work exit (economic inactivity or (early) retirement) died.	Multistate model biased on monthly transitions. Period data. Censoring at age of 66.	WLE between ages 50–66 by gender and education. The expected time in other states by gender and education is also shown.
***Occupational class differences***					
Dudel et al. 2018 [4]	15–99 years old individuals who pay taxes or receive social security benefits; *N* = 1 272 695: 2004–2013; Spain	Spanish Continuous Working Life Sample, register-based social security benefits. Eurostat life tables based on the EU Labor Force Survey; Registers.	Employed, unemployed, retired, inactive, died or age 99 years.	Multistate Markov model based on annual transitions. Period data. No age censoring.	Remaining LE in employment at age 15, 2004 to 2012 by gender and occupational class. Remaining LE at age 15 in other labour force state by gender, occupational class and year is also shown.
Leinonen et al. 2018 [5]	50–69 years old; *N* = 5 170 689 unweighted person-years; 1988–2007; Finland	Administrative registers with annual information through the end of 2012; Registers.	Employment, unemployed, disability retirement, other forms of early retirement, statutory retirement, outside of the labor force.	Sullivan method. Period and cohort data. No age censoring	Working and retirement LE at age 50 by gender and social class.
Lorenti et al. 2019 [45]	15–99 years old; *N* = 880 000; 2003–2013; Italy	Nationally representative Longitudinal Sample INPS (National Institute of Social Security); Registers.	Employed, unemployed, retired, economically inactive, died.	Multistate Markov model based on annual transitions. Period data. No age censoring	Remaining LE in employment (WLE) at age 15 for years from 2003 to 2013 by gender and occupational class.
Schram et al. 2021 [50]	50–63 years old; *N* = 415 105; 2004–2014; Finland	Nationally representative register-based cohort; Registers.	Work, time-restricted work disability, unemployment, economic inactivity, disability retirement, retirement, died.	Multistate Cox regression model based on daily transitions. Cohort data. Censoring at age of 63.	WLE between ages 50–63 by gender and occupational class. The expected time in other states by gender and occupational class is also shown.
Lynch et al. 2022 [51]^e^	50–102 years old; *N* = 15 284; 2002–2013; U.K.	The English Longitudinal Study of Ageing, data from six cross-sectional samples; Self-reports.	Healthy and in work, not healthy and in work, healthy and not in work, not health and not in work, died.	Interpolated Markov chain multistate modelling of panel data using multinomial logistic regression based on annual transitions. Period data. No age censoring.	WLE (healthy and in work, and not healthy and in work), healthy life expectancies (healthy and in work and healthy not in work, HLE), at age 50. HWLE for the 50–75 age range by occupational class.
***Educational and occupational class differences***					
Hayward & Grady 1990 [36]^a^	55 years old men in the labour force; *N* = 2 816; 1966–1983; U.S.	The National Longitudinal Survey of Older Men; Self-reports.	In the labour force, retirement, disability, died.	Increment–decrement model with transition rates derived from multivariate hazard models for each type of transition. Cohort data. No age censoring.	WLE at age 55 by education and occupation.
Parker et al. 2020 [21]^e^	50–102 years old; *N* = 15 284; 2002–2013; U.K	The English Longitudinal Study of Ageing, data from six waves of cross-sectional samples; Self-reports.	Healthy and in work, not healthy and in work, healthy and not in work, not health and not in work died.	Interpolated Markov chain multistate modelling of panel data using multinomial logistic regression based on annual transitions. Period data. No age censoring.	LE, HLE (healthy and in work and healthy not in work), WLE (healthy and in work, and not healthy and in work) and healthy WLE (HWLE) at age 50 by education and occupational class.
Dudel et al. 2021 [48]	55–64 years old (born 1941 and 1955); 1996–2019; Germany	The annual German Microcensus survey; Self-reports	Employed, unemployed retired/inactive.	Modified Sullivan’s method. Cohort data. Censoring at age of 65.	WLE adjusted for working time (measured in full-time equivalent years) for the 55 to 64 age range by region, gender, education and occupational class.

*Abbreviations: U.S. *United States, *U.K. *United Kingdom, *WLE *Working life expectancy, *LE *Life expectancy, *HLE *Healthy life expectancy, *HWLE *Healthy working life expectancy, *SHARE* Survey- survey of Health, Ageing and Retirement in Europe

^a^Reports from the same study - The National Longitudinal Survey of Older Men

^b^Reports from the same study - Bureau of Labour Statistics and Current Population Surveys 1990–2000

^c^Reports from the same study - Current Population Survey (U.S)

^d^Reports from the same study - The Health and Retirement Study (HRS)

^e^Reports from the same study - The English Longitudinal Study of Ageing

The WLE or related measures were estimated using self-reports in 19 reports and register data in seven reports (Table 2). WLE was most frequently (*n* = 15) defined as employment expectancy. It was defined as economic activity expectancy (including employment and unemployment) in eight reports and as productive work expectancy (employed and not being e.g., on sickness absence or in subsidized employment) in two reports. In the vast majority of the reports (*n* = 21), the incidence-based approach was used for estimation of WLE. Only one report [50] used continuous transitions, a few reports used monthly transitions, while in the remaining reports, data were interval-censored with one or more years between the transitions. One report performed a simulation of life trajectories based on estimated hazard rates for mortality, employment, and retirement [39]. Five reports presented socioeconomic differences in HWLE. Half of the reports also explored socioeconomic differences in WYL. The vast majority of the included reports (*n* = 21; 81%) examined educational differences in WLE or related indicators, while fewer reports (*n* = 7) examined social/occupational class differences. No reports on income differences in WLE or related measures were found.

**Table 2:** Overall characteristics of the 26 included reports

Characteristics		Number of reports	References
**Data source**			
Self-reports		19	[1, 2, 21, 24, 34, 36–48, 51]
Registers		7	[4–6, 30, 49, 50, 52]
**Gender stratification**			
Only men		2	[36, 37]
Only women		1	[44]
Gender stratification		20	[1, 2, 4–6, 30, 34, 38–43, 45–50, 52]
No gender stratification		3	[21, 24, 51]
**WLE (definition)**			
Economic activity expectancy (employed or unemployed)		8	[1, 2, 36, 37, 41, 43, 44, 52]
Employment expectancy		13	[4–6, 21, 30, 34, 38, 40, 45–48, 51]
Productive work expectancy		2	[49, 50]
**HWLE (definition)**		5	
Years being healthy and employed		3	[21, 39, 51]
Years employed without disability		1	[24]
Years being economically active without functional limitations		1	[1]
**Working years lost**		13	
Retirement		6	[5, 6, 34, 36, 37, 46]
Disability		6	[5, 6, 36, 37, 47, 50]
Unemployment		6	[4–6, 42, 46, 50]
Economically inactive/outside of the labour market		10	[4–6, 34, 42, 44, 46, 47, 50]
Involuntary exit (unemployment, disability)		1	[30]
Voluntary exit (retirement, inactive)		1	[30]
**Socioeconomic indicator**			
Level of education		21	[1, 2, 6, 21, 24, 30, 34, 36–44, 46–49, 52]
Social or occupational class		7	[4, 5, 21, 45, 48–50]
**Approach for calculation of WLE**			
Prevalence-based		5	[1, 2, 5, 46, 48]
Incidence-based		21	[4, 6, 21, 24, 30, 34, 36–45, 47, 49–52]
**Type of life Table**^**a**^			
Period		21	[1, 2, 4–6, 21, 30, 34, 38–47, 49–52]
Cohort		6	[5, 24, 36, 37, 48, 50]
**Interval-censoring**^**b**^			
No interval censoring		1	[50]
Less than one year		4	[6, 30, 42, 49]
One year or more		16	[4, 21, 24, 34, 36–41, 43–45, 47, 49–52]
**Censoring by age**			
Around retirement age		10	[1, 2, 6, 24, 30, 42, 48–50, 52]
No age censoring		16	[4, 5, 21, 34, 36–41, 43–47, 51]

^a^In study by Leinonen and co-authors (2018) both types of life table were used

^b^Only for studies that used incidence-based approach for estimation of WLE or related indicators

Five reports did not include numerical results for specific socioeconomic categories and were excluded from the data synthesis [2, 40, 47–49]. A study by Tetzlaff and co-authors [52] was excluded because it utilized very specific data of a particular region of Germany as well as of a particular health insurance.

#### Educational differences

**Fig. 2:**
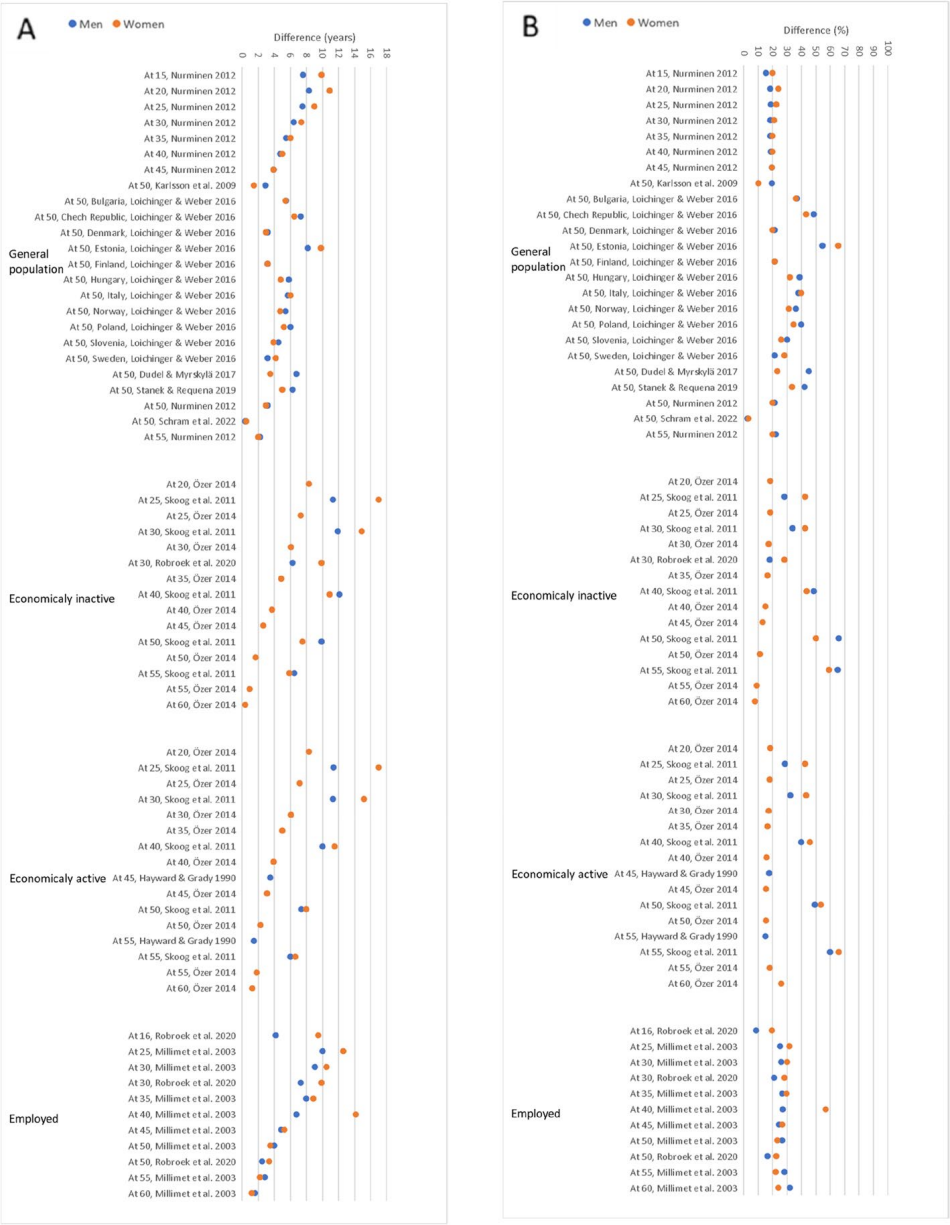
Educational differences in WLE among men and women. **A** Absolute differences (years); **B** Relative differences (share of remaining working life)

All studies found a longer WLE among persons with high education compared to those with low education (Supplementary Table S3). Most of the studies observed a larger absolute educational difference in WLE at ages between 15 and 40 years among women than men (Fig. 2A). Gender gap in the educational differences in WLE after age of 45 was relatively small. Across all ages, absolute educational differences in WLE tended to be smaller among initially employed than initially economically active (including both employed and unemployed) or inactive individuals (Fig. 2A).

In most of the studies, relative differences between persons with high and low education were somewhat similar between men and women irrespective of the initial labour market state and age at which WLE was estimated (Fig. 2B). As compared with high-educated persons, working life years of low-educated persons were expected to be, on average, 30% shorter among men and 27% among women. The magnitude of differences varied noticeably by definition used for WLE and study population, particularly among women (Table 3). Studies with WLE defined as economic activity expectancy (including both employment and unemployment) reported the largest educational difference (mean 38% among men and 41% among women). In such studies, differences in the general population were smaller than in populations initially economically active or inactive. Studies with WLE defined as productive work expectancy (time expected to be at work) reported the smallest educational differences (mean 11% among men and 18% among women).

**Table 3:** Influence of WLE operationalization on educational differences in WLE among men and women. Mean and median relative difference (share of remaining working life)

		Men		Women	
Definition of WLE	Population	Mean (%)	Median (%)	Mean (%)	Median (%)
All combined	Total	30	27	27	22
Economic activity expectancy					
	Total	38	38	41	42
	General population	35	37	34	31
	Initially active^a^	48	49	47	47
	Initially inactive^b^	38	32	50	46
Employment expectancy					
	Total	24	21	20	20
	General population	23	19	21	20
	Initially active			14	15
	Initially inactive			18	17
	Initially employed	26	27	30	28
Productive work expectancy	Total	11	17	18	23
	Initially employed	14	17	24	23

^a^Initially economically active (employed or unemployed)

^b^Initially economically inactive (retired, studied or those in household and care activities)

The magnitude of educational differences between incidence- and prevalence-based methods was similar. However, studies using incidence-based methods based on data with short censoring intervals (i.e., less than a year) reported smallest educational differences. Studies which used cohort life tables for calculations of WLE, tended to report smaller educational differences than studies which used period life tables.

Differences in WLE at age 50 between high and low-educated persons, which were most examined, varied considerably across the studies (from 0.40 to 9.90 years among men and from 0.50 to 9.80 among women). On average, WLE at age 50 among low-educated persons was 5.1 (men) and 4.6 years (women) shorter than among high-educated persons (median values 5.4 and 4.4 years among men and women, respectively). The smallest educational differences (0.40 and 0.50 among men and women, respectively) were reported in a general population study [30], where WLE was defined as years expected to be in paid employment and estimated using incidence-based approach with monthly transitions between the labour market states.

#### Occupational class differences

**Fig. 3:**
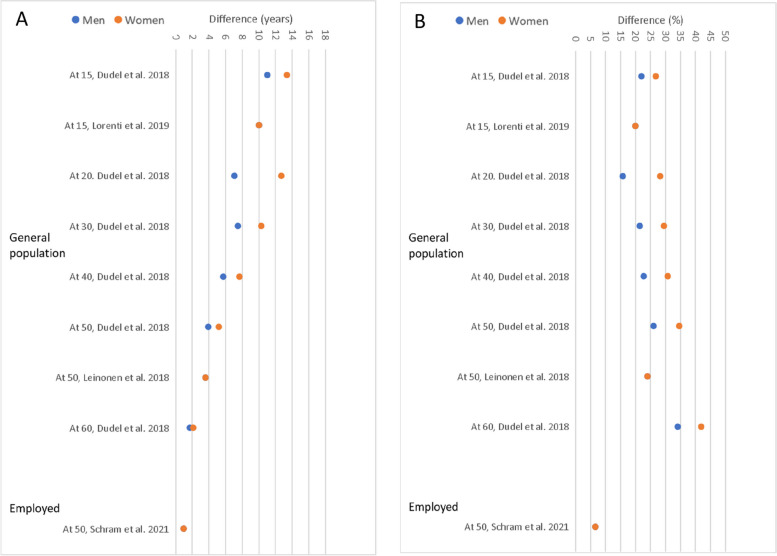
Occupational class differences in WLE among men and women. **A** Absolute differences (years); **B** Relative differences (share of remaining working life)

Occupational class differences were found in all studies, and individuals in non-manual occupations had longer WLE than individuals in manual occupations (Supplementary Table S3). Larger occupational class differences in WLE among women than men were found in the study by Dudel and co-authors [4], while in the other studies, differences were similar in both genders (Fig. 3A and B). As compared with persons belonging to high occupational class, working life years of persons in low occupational class was expected to be, on average, shorter by 21% (median: 22%) among men and 27% (median: 28%) among women.

The smallest occupational class differences were found in the study of initially employed men and women, where incidence-based approach and continuous scheme of observations (data were not interval-censored) was used for estimation of WLE [50].

#### Socioeconomic differences in healthy working life expectancy

Two studies examined educational differences in both WLE and HWLE. Smaller educational differences in HWLE than in WLE at age 50 (3.50 vs. 3.80 years) were found in a study by Parker et al. [21]. While according to another study [24] educational differences in HWLE at age 58 were much larger than in WLE, being 1.4 and 0.8 years, respectively (Supplementary Table S3). Larger occupational class differences in HWLE than in WLE at age 50 were found, being 1.60 and 1.40 years, respectively [21] (Supplementary Table S3).

#### Socioeconomic differences in working years lost

Six out of 13 studies that explored socioeconomic differences in WYL presented results for WYL due to unemployment, disability and early retirement (Table 2, Supplementary Table S4). All studies found that irrespective of age low-educated persons are expected to lose more working years due to unemployment and disability retirement than high-educated persons. (Supplementary Figure S1, Supplementary Table S4). However, the opposite phenomenon was seen for WYL due to early retirement. Overall, in both genders, educational differences for WYL due to unemployment were larger than for disability retirement. Educational differences in WYL due to unemployment at age 15–40 years were on average 0.6 years larger among women than men (vary from 0.8 to 7.4 years and from 0.7 to 5.7 years in women and men, respectively). In contrast, Robroek and co-authors [6] found larger educational differences in WYL due to disability retirement among men than women.

All studies found that irrespective of age, persons belonging to low occupational class are expected to lose more working years due to unemployment and disability retirement than persons in high occupational class (Supplementary Figure S2, Supplementary Table S4). Both men and women in low occupational class at age 50 were expected to lose about one year more than persons belonging to high occupational class due to unemployment.

## Discussion

Our main findings reveal that irrespective of socioeconomic indicator, persons with low socioeconomic position have shorter WLE than those with high socioeconomic position. On average, WLE in persons with low education is 29% (men) and 27% (women) shorter than in those with high education. The magnitude of educational differences varies noticeably depending on the definition used for WLE and study population. Overall, the occupational class differences in WLE were more pronounced among women than men (with mean difference being 27% vs. 21%). Among low-educated persons more working years were lost due to unemployment and disability retirement but less due to other types of non-employment than among high-educated persons. Moreover, educational differences in WYL due to unemployment were larger than due to disability retirement.

In general, WLE represents the average expected working life duration for individuals at a specific age. Different definitions of WLE are used in the literature to capture the dynamic patterns of entering, exiting and re-entering employment during the lifespan and to distinguish between employment and non-employment labour market states, as well as healthy and unhealthy working life. For this review, we included the following most commonly used definitions: economic activity expectancy (expected years in either paid employment or unemployment), employment expectancy (expected years in paid employment), productive work expectancy (expected years in paid employment, excluding sickness absence) and healthy WLE (expected years in working life while being in “good health”).

The direction of socioeconomic differences in WLE that we found, was expected, knowing socioeconomic inequalities in labour market attachment, health and life expectancy exist [53–56]. However, we observed a noticeable variation in the magnitude of educational differences in WLE across the studies. One of the reasons for the large variation in the educational differences is the variation in methods to estimate WLE. WLE is a probabilistic construct, estimated using multistate models based on ether period or cohort life tables, of which the first one is most frequently used. WLE, building on life tables for a given period (one year or several years), describes patterns of labour market attachment in a synthetic or hypothetical cohort with an assumption that the age-specific mortality and participation rates in different labour market states during remaining years will be the same as those observed in that period. This is a rather strong assumption, which might be violated and not necessary be realizable similarly across different study populations, age groups, as well as time periods. The larger the deviation from the assumption is, the higher the likelihood of bias in the WLE estimates will be. Furthermore, the direction of bias might be different for different subgroups of the study population. At younger age the WLE is likely overestimated, while at older age it is likely underestimated. For people below age of 30 years, high heterogeneity in labour market participation of people increases uncertainty for the estimation of WLE.

The studies included into our review were very heterogeneous with regard to factors that may influence the socioeconomic differences in WLE (e.g., operationalization of WLE, study population, categorization of socioeconomic factors, methods of estimation of WLE). Due to above mentioned reasons the absolute socioeconomic differences in WLE across different ages as well as different study populations or subgroups are not comparable. As such, in the current review we used relative socioeconomic differences to better understand the reasons for the large observed variation in the socioeconomic differences in WLE across the studies.

We found that studies with WLE defined as economic activity expectancy reported the largest differences, while the smallest differences were found for productive work expectancy. WLE defined as economic activity expectancy covers both employment and unemployment and thus will result in higher estimates of the WLE, as compared with narrower definitions.

Large variation in the reported socioeconomic differences can also be attributed to the stage of economic cycle when WLE was estimated/calculated. Educational differences might be intensified during the economic crisis since less-educated persons are more vulnerable to unemployment than higher-educated persons [34, 57]. Unemployment, in particular long-term unemployment, is increasing during economic recession. Dudel and co-authors [4] examined the influence of economic crisis on WLE in Spain and found a tremendous effect, which differed largely by gender and occupational category. Among unskilled manual workers, the average proportion of lifetime spent in unemployment and outside the labour market, increased markedly during the economic crisis, while it remained at the same level among people in skilled non-manual occupations.

Knowing that health is an important contributor to earlier permanent withdrawal from the labour market [13, 58], decomposition of WLE to healthy and unhealthy WLE is warranted. A recent study in 14 countries within the Organisation for Economic Co-operation and Development, found noticeable cross-country variation in trends of HWLE between 2002 and 2017, while an increasing trend in unhealthy WLE was observed in most of the countries [22]. Five reports on HWLE from three studies were included in the current review. All studies observed longer HWLE at age 50 and later among individuals with high socioeconomic position than among those with low socioeconomic position. However, the magnitude of the differences varied across the studies.

An association of income level with morbidity and mortality is well documented [59–61]. Since 2001, the income-related health gap widened substantially in most of the western countries due to the faster increase in life expectancy among individuals with higher incomes than those with lower incomes [62, 63]. How income influences working life duration is poorly understood, since no reports on the association of income with either WLE or HWLE was captured by our searches. The lack of reports on income inequality in WLE might be partly due to the fact that income is much more volatile than education or occupational class.

There are several methodological choices and challenges in examining socioeconomic differences in WLE. Most importantly, the levels of WLE and inequalities in WLE differ depending on the study population or sub-populations where it is estimated; depending on whether a cohort perspective or a period perspective is used; and depending on whether prevalence-based or incidence-based methods are applied.

For example, results on educational differences in WLE at age 50 from two Dutch studies were very different. One study [30] found that WLE at age 50 in low-educated people was by 0.4 (men) and 0.5 (women) years shorter than in high-educated people. The corresponding numbers in another study [6], were 2.5 and 3.4 years in men and women, respectively. The study population of the former study consisted of around 12 000 participants of the online STREAM cohort. While the latter study utilized nationally representative data from Statistics Netherlands on about 5 million individuals. Otherwise, both studies were similar with regard of WLE definition and estimation method, as well as educational categories. Similarly, occupational class differences in WLE at age 50 varied in two Finnish studies. In one study, in both genders the WLE at age 50 among manual workers was 3.6 years shorter than among upper-level non-manual workers [5]. In another study, the difference in WLE between manual and upper-level non-manual workers was only 1 year [50]. The two studies differed with regard to study population (general vs. employed), WLE definition (employment vs. productive work expectancy) and method of estimation (prevalence- vs. incidence-based). The occupational class differences in the second study [50] are likely underestimated due to healthy worker effect. Manual workers are more likely to leave the labour force before age of 50 years due to reduced workability than the upper-level non-manual workers. The examples presented above suggest that the socioeconomic differences in WLE at age of 50 might be attenuated due to selection bias in the study population.

Dudel and Myrskylä [35] found that the same data can show increases in WLE in the period perspective but stagnation or decline of WLE in the cohort perspective. The increase in period WLE was caused by an increase in employment rates in the most recent years of the data; however, this increase did not compensate for reductions in employment rates some birth cohorts experienced in earlier periods, leading to the decrease in the cohort perspective. Incidence-based methods are known to capture sudden changes in employment better than prevalence-based methods which is particularly relevant if the data used in an analysis includes a macroeconomic shock like a recession (e.g., Dudel et al. [4]).

Moreover, we found that levels of inequalities also depend on whether period or cohort WLE is estimated. This is due to the fact that period WLE can amplify group differences, in particular if there are macroeconomic shocks. This is because period WLE for a period affected by a shock implicitly assumes that individuals are exposed to the resulting adverse economic conditions throughout their whole life, while real cohorts usually only experience these conditions for a few years. Moreover, members of real cohorts might try to compensate for years of working life lost once economic conditions improve (e.g., they might extend time of old age retirement due to less pension savings) while this is not captured in period WLE. However, the direction of the inequalities found in the literature is rather consistent irrespective of the specific methods used.

### Strengths and limitations

To our knowledge, the present study is the first to review the literature on educational and occupational class differences in WLE and HWLE. We believe that our search strategy allowed us to broadly capture the relevant scientific reports on this topic. We used a standard data extraction form for each report included in the scoping review, thus our summarized information should be as robust and standardized as possible. However, the current review also has limitations. Due to heterogeneity of included reports and large differences in the country context which influenced the results, we were neither able to conduct a systematic review nor a meta-analysis of the findings. Instead, we aimed to overview which different operationalizations of WLE, as well as socioeconomic indicators, were used in the literature and identify the methodological challenges in analysing the socioeconomic differences in WLE. In particular, we examined whether the magnitude of socioeconomic differences varies by the used indicators, by different study populations or according to different definitions of WLE and approaches used for its estimation. We focused on WLE and did not include measures of the length of working life which do not account for all possible labour market transitions; one example of such a a measure is the effective retirement age [64, 65].

### Future research needs

There is a need for further research on several aspects of WLE. First, the majority of studies focuses on a single country, and only very little comparative research is available (e.g., [1]). Such research is essential for understanding how different institutional contexts and policy regimes are shaping WLE and potentially influencing socioeconomic inequalities in WLE. However, differences in the distribution of socioeconomic position across the countries challenge comparability of results. It could be of interest to use inequality measures, such as relative index of inequality and the slope index of inequality, for comparisons of the socioeconomic gradient in WLE.

Second, almost all studies on WLE are rather descriptive, with only a few exceptions. This means that the drivers and causes of trends and inequalities in WLE are poorly understood. There is no “golden standard” regarding operationalization of WLE and estimation methods, which are typically selected based on data availability. Several factors may cause uncertainty in the estimates, with the direction of bias (overestimation or underestimation) changing and depending on specific circumstances and interplay of those factors. Therefore, future studies should pay attention to more detailed reporting about study population, institutional context, operationalization of WLE, analytical approaches for estimation and underlying assumptions.

Studies which are connecting WLE to health and working conditions have emerged recently [66], starting to fill this gap. Nevertheless, more research is needed. The study of WLE could be further extended to include characteristics of employment, such as full- vs. part-time or the quality of work [67]. Finally, most studies focus on a rather wide age range. Some studies have shown that there are specific years which are particularly vulnerable. For instance, Dudel et al. [4] provide evidence that young Spanish workers below age 30 were particularly affected by the financial crisis in 2007/8. Identifying such vulnerable groups of workers will help design better targeted labour market and pension policies. Most of the studies included into this review examined educational differences in WLE, while no studies on income differences were found. Furthermore, socioeconomic differences in HWLE are largely unknown. Future research is needed to fill the knowledge gap on HWLE and potential influences of income on working life duration.

## Conclusions

This scoping review adds to the literature in several ways. We provide the first review of socioeconomic inequalities in WLE and HWLE. Our results show that disparities between socioeconomic groups are often substantial, and persistent over time and across countries. Moreover, our results show a large variability in the levels of inequalities, depending on the age at which WLE is measured; the operationalization of WLE (including definition and estimation method) and socioeconomic position; the institutional context and the examined study population.

The directions of the inequalities are, however, largely consistent across studies. That is, higher-educated individuals tend to have longer WLE than lower-educated individuals, and individuals in non-manual occupations work longer than individuals in manual occupations. Overall, our findings show that despite these consistencies, some caution is advisable when comparing studies of WLE. The inequalities we report are highly relevant for policy makers and pose serious challenges to equitable retirement and pension policies. Many policy interventions aimed at increasing the length of working life follow a one-size-fits-all approach, which does not take these inequalities into account.

### Supplementary Information

**Supplementary Material 1.**

## Data Availability

All data relevant to the study are included in the article or uploaded as supplementary information.

## Abbreviations

WLE: Working life expectancy
HWLE: Healthy working life expectancy
WYL: Working years lost
