# Supplementary material for: Socioeconomic differences in working life expectancy: a scoping review
Source: BMC Public Health. 2024 Mar 7;24:735. doi: 10.1186/s12889-024-18229-y (PMC10921693; doi:10.1186/s12889-024-18229-y)
Supplement: Supplementary file 1 — Supplementary Material 1. [file 12889_2024_18229_MOESM1_ESM.docx]

**Supplementary material**

Table S1. Excluded reports and reason for exclusion.

| **Author, year** | **Reason for exclusion** |
| --- | --- |
| Liefbroer et al. 1999 | Duration of working life, not working life expectancy (WLE) |
| Järvholm et al. 2014 | Retirement age, not WLE |
| Ramamurthy et al. 2015 | No results on WLE by education or occupational class were reported |
| Kang et al. 2016 | Retirement age, years lost due to retirement, not WLE |
| de Wind A et al. 2018 | No results on WLE by education or occupational class were reported |
| Laaksonen et al. 2018 | Retirement age, not WLE |
| Qi et al. 2018 | Retirement age, not WLE |
| Kadefors et al. 2019 | Retirement age, not WLE |
| Kuivalainen et al. 2020 | Age at retirement, not WLE |
| Quinby & Wettstein 2021 | Expected additional years of work ability, not WLE |
| Heller et al. 2022 | No results on WLE by education or occupational class were reported |
| Riekhoff et al. 2022 | Effective exit ages, not WLE |

**References**

1. Liefbroer AAT C, Liefbroer AC, Henkens K. Labour market careers of successive cohorts of older men in the Netherlands: Changes in age at retirement and in length of working lives. Genus. 1999;55:101-19.
2. Järvholm B, Stattin M, Robroek SJW, Janlert U, Karlsson B, Burdorf A. Heavy work and disability pension - A long term follow-up of Swedish construction workers. Scand J Work Environ Health. 2014;40:335-42.
3. Ramamurthy S, Sedgley N. Human Capital Choice and the Wage Gap: The Role of Worklife Expectancy and Statistical Discrimination. J Labor Res. 2015;36:175-87.
4. Kang YJ, Kang MY. Chronic Diseases, Health Behaviors, and Demographic Characteristics as Predictors of Ill Health Retirement: Findings from the Korea Health Panel Survey (2008-2012). PLoS One. 2016;11:e0166921.
5. de Wind A, van der Noordt M, Deeg DJH, Boot CRL. Occup [Working life expectancy in good and poor self-perceived health among Dutch workers aged 55-65 years with a chronic disease over the period 1992-2016.](https://pubmed.ncbi.nlm.nih.gov/30194272/)  Occup Environ Med. 2018;75:792-7.
6. Laaksonen M, Rantala J, Järnefelt N, Kannisto J. Educational differences in years of working life lost due to disability retirement. Eur J Public Health. 2018;28:264-8.
7. Qi, HD, Helgertz, J, Bengtsson, T. Do notional defined contribution schemes prolong working life? Evidence from the 1994 Swedish pension reform. J Economics Ageing. 2018;12:250-67.
8. Kadefors R, Nilsson K, Ostergren PO, Rylander L, Albin M. Social inequality in working life expectancy in Sweden. Z Gerontol Geriatr. 2019; 2:52-61.
9. Kuivalainen S, Nivalainen S, Järnfelt N, Kuitto K. Length of working life and pension income: empirical evidence on gender and socioeconomic differences from Finland. J Pension Econom & Finance .2020;19:126-46.
10. Quinby L, Wettstein G. Are Older Workers Capable of Working Longer? Center for Retirement Research (CRR) Working Paper WP 2021-8, June 2021. Available at SSRN: https://ssrn.com/abstract=3936027 or <http://dx.doi.org/10.2139/ssrn.3936027>
11. Heller C, Sperlich S, Tetzlaff F, Geyer S, Epping J, Beller J, et al. Living longer, working longer: analysing time trends in working life expectancy in Germany from a health perspective between 2002 and 2018. Eur J Ageing. 2022;19:1263-76.
12. Riekhoff AJ, Kuitto K. Educational differences in extending working lives. Trends in effective exit ages in 16 European countries. Helsinki; 2022: Finnish Centre for Pensions, Finnish Centre for Pensions Studies no 02/2022 Available at: https://urn.fi/URN:ISBN:978-951-691-345-5 (Accessed on 10.07.2023)

Table S2. Duplicated reports from the same study.

| Study | Report 1 | Report 2 | Included into data synthesis |
| --- | --- | --- | --- |
| The National Longitudinal Survey of Older Men | Hayward & Grady 1990 | Hayward & Lichter 1998 | Hayward & Grady 1990 |
| Buereau of Labour Statistics and Current Population Surveys 1990-2000 | Millimet et al. 2003 | Millimet et al. 2010 | Millimet et al. 2003 |
| Current Population Survey (U.S) | Skoog et al. 2011 | Krueger & Slesnick 2014 | Skoog et al. 2011 |
| The Health and Retirement Study (HRS) | Dudel & Myrskylä 2017 | Lorenti et al. 2020 | Dudel & Myrskylä 2017 |
| The English Longitudinal Study of Ageing | Parker et al. 2020 | Lynch et al. 2022 | Parker et al. 2020 |

**References**

Hayward MD, Grady WR. Work and retirement among a cohort of older men in the United States, 1966-1983. Demography. 1990;27:337-56.

Hayward MD, Lichter A life cycle model of labour force inequality. Extending Clogg’s life table approach. Soc Meth Res. 1998:26:480-510.

Millimet DL, Nieswiadomy M, Ryu H, Slottje D. Estimating worklife expectancy: an econometric approach. J Econometrics 2003;113:83-113.

Millimet D L, Nieswiadomy M, Slottje DJ. Detailed estimation of worklife expectancy for the measurement of human capital: accounting for marriage and children. J Econom Surveys 2010; 24(2):339-61.

Skoog GR, Ciecka JE, and Krueger KV. The Markov Process Model of Labor Force Activity: Extended Tables of Central Tendency, Shape, Percentile Points, and Bootstrap Standard Errors. J Forensic Economics. 2011;22:165-229.

Krueger KV, Slesnick F. Total worklife expectancy. J Forensic Economics. 2014;25:51-70.

Dudel C, Myrskylä M. Working Life Expectancy at Age 50 in the United States and the Impact of the Great Recession. Demography 2017; 54:2101-23.

Lorenti A, Dudel C Hale JM, Myrskylä M. Working and disability expectancies at older ages: The role of childhood circumstances and education. Social Science Research 2020; 91:102447. (<https://reader.elsevier.com/reader/sd/pii/S0049089X20300454?token=97D1F990708AC40653AD5CD1E10460E835A2A25E440A5EFC4DAC89CF2A3EB611E8973384DEBADDD1C1B1F825101928E7&originRegion=eu-west-1&originCreation=20220819113533>)

Lynch M, Bucknall M, Jagger C, Wilkie R. Healthy working life expectancy at age 50 for people with and without osteoarthritis in local and national English populations. Sci Rep 2022; 12(1):2408.

Parker M, Bucknall M, Jagger C, Wilkie R. Population-based estimates of healthy working life expectancy in England at age 50 years: analysis of data from the English Longitudinal Study of Ageing. Lancet Public Health 2020; 5(7):e395-e403.

Table S3. Results from the included studies on socioeconomic differences in WLE and HWLE

| Author, year, country | Study population | Operationalization of socioeconomic factor | Definition of WLE | Age | Results |
| --- | --- | --- | --- | --- | --- |
| Hayward & Grady 1990;  U.S. | In the labour force | Education:  < 12 years (low, L);  13-15 years (medium, M);  > 16 years (high, H) | Economic activity expectancy | Between 55-70 | **Men:** 8.4 (L), 9.4 (M), 9.9 (H). |
| Hayward & Lichter 1998;  U.S. | In the labour force | Education:  less than high school (< 12 years, low, L);  more than high school (>16 years, high, H). | Economic activity expectancy | Between 45-75 | **Men, with reentry:** 16.4 (L), 19.9 (H);  **Men, without reentry:** 11.2 (L), 14.6 (H) |
|  |  |  |  | Between 55-75 | **Men, with reentry:** 8.1 (L), 11.1 (H);  **Men, without reentry:** 4.7 (L), 6.7 (H) |
| Millimet et al. 2003;  U.S. | In the labour force | Education:  less than high school degree (low education, L);  a high school degree (medium, M);  college or above (high, H) | Employment expectancy | At age 30 | Initially employed  **Men:** 22.1 (L), 28.1 (M), 31.2 (H);  **Women:** 17.3 (L), 24.7 (M), 27.7 (H) |
|  |  |  |  | At age 40 | Initially employed  **Men:** 15.3 (L), 19.7 (M), 22.1 (H);  **Women:** 12.8 (L), 17.6 (M), 20.0 (H) |
|  |  |  |  | At age 50 | Initially employed  **Men:** 9.5 (L), 11.9 (M), 13.5 (H);  **Women:** 8.5 (L), 10.6 (M), 12.0 (H) |
| Karlsosn et al. 2009;  UK | General population | Education:  low education (L); medium education (M);  high education (H) | Employment expectancy | At age 50 | General population  **Men:** 13.5 (L), 13.6 (M), 16.4 (H);  **Women:** 10.3 (L), 11.0 (M); 12.8 (H);  Employed  **Men:** 14.8 (L), 14.8 (M), 17.3 (H);  **Women:** 12.1 (L), 12.4 (M), 14.2 (H);  Non-employed  **Men:** 4.5 (L), 4.2 (M), 7.1 (H);  **Women:** 3.3 (L), 3.4 (M), 4.9 (H) |
| Skoog et al. 2011; U.S. | General population | Education:  eight groups  (1) < 12 years of education but no high school diploma or GED,  (2) GED but no high school diploma,  (3) high school diploma but no college,  (4) some college but no degree,  (5) associate’s degree but no bachelor’s degree,  (6) bachelor’s degree but no master’s or higher degree,  (7) master’s degree but no professional degree or PhD, and  (8) professional degree or PhD. | Economic activity expectancy | Between 25- 75 | Initially active  **Men:** 29.4 (1), 29.9 (2), 33.6 (3), 34.1 (4), 35.3 (5), 36.5(6), 38.6 (7), 40.8 (8);  **Women:** 20.7 (1), 25.2 (2), 28.7 (3), 31.2 (4), 33.6 (5), 33.2 (6), 34.9 (7), 37.7 (8);  Initially inactive  **Men:** 27.3 (1), 28.2 (2), 31.7 (3), 32.6 (4), 33.7 (5), 35.2 (6), 37.2 (7), 38.6 (8);  **Women:** 18.8 (1), 23.2 (2), 26.4 (3), 29.5 (4), 31.9 (5), 31.4 (6), 32.7 (7), 35.8 (8) |
|  |  |  |  | Between 30- 75 | Initially active  **Men:** 25.5 (1), 26.4 (2), 29.6 (3), 30.0 (4), 31.0 (5), 32.1 (6), 34.1 (7), 36.8 (8);  **Women:** 22.7 (1), 23.6 (2), 27.2 (3), 28.2 (4), 29.0 (5), 30.7 (6), 32.2 (7), 34.6 (8);  Initially inactive  **Men:** 18.4 (1), 22.2 (2), 25.5 (3), 27.5 (4), 29.6 (5), 29.3 (6), 31.0 (7), 33.6 (8);  **Women:** 16.2 (1), 20.1 (2), 22.7 (3), 25.4 (4), 27.1 (5), 26.4 (6), 28.3 (7), 31.1 (8) |
|  |  |  |  | Between 40- 75 | Initially active  **Men:** 17.7 (1), 19.0 (2), 21.0 (3), 21.3 (4), 22.2 (5), 22.9 (6), 25.0 (7), 27.7 (8);  **Women:**13.7 (1), 16.5 (2), 18.8 (3), 20.3 (4), 21.6 (5), 21.8 (6), 23.0 (7), 25.2 (8);  Initially inactive  **Men:**13.8 (1), 15.1 (2), 17.8 (3), 18.2 (4), 19.2 (5), 20.5 (6), 23.2 (7), 25.9 (8);  **Women:** 10.7 (1), 13.3 (2), 15.5 (3), 17.1 (4), 18.6 (5), 18.4 (6), 19.7 (7), 21.6 (8) |
|  |  |  |  | Between 50- 75 | Initially active  **Men:**11.4 (1), 11.8 (2), 13.3 (3), 13.3 (4), 14.0 (5), 14.1 (6), 16.0 (7), 18.8 (8);  **Women:**8.9 (1), 10.5 (2), 12.1 (3), 13.0 (4), 13.7 (5), 14.0 (6), 14.6 (7), 16.9 (8);  Initially inactive  **Men:** 6.1 (1), 7.6 (2), 8.9 (3), 8.8 (4), 9.7 (5), 10.1 (6), 13.4 (7), 16.0 (8);  **Women:** 5.1 (1), 6.1 (2), 7.6 (3), 9.1 (4), 9.7 (5), 10.1 (6), 10.4 (7), 12.6 (8) |
|  |  |  |  | Between 55- 75 | Initially active  **Men:** 8.6 (1), 8.9 (2), 9.7 (3), 9.9 (4), 10.4 (5), 10.2 (6), 11.9 (7), 14.6 (8);  **Women:** 6.7 (1), 8.0 (2), 9.0 (3), 9.7 (4), 10.1 (5), 10.3 (6), 10.7 (7), 13.3 (8);  Initially inactive  **Men:** 3.8 (1), 4.4 (2), 5.2 (3), 5.3 (4), 5.4 (5), 5.8 (6), 8.1 (7), 10.3 (8);  **Women:**3.0 (1), 3.5 (2), 4.3 (3), 5.4 (4), 6.0 (5), 6.0 (6), 6.4 (7), 8.9 (8) |
| Nurminen 2012;  Finland | General population | Education:  primary or lower secondary education (ISCED levels 1–2), (low, L);  secondary education (ISCED  levels 3–4), (medium, M); tertiary (ISCED  levels 5–6), (high, H) | Employment expectancy | Between 25-60 | **Men:** 26.6 (L), 30.1 (M), 34.1 (H);  **Women:** 23.2 (L), 29.0 (M), 32.2 (H) |
|  |  |  |  | Between 30-60 | **Men:** 23.3 (L), 26.1 (M), 29.7 (H);  **Women:** 20.9 (L), 25.5 (M), 28.3 (H) |
|  |  |  |  | Between 40-60 | **Men:** 15.6 (L), 17.3 (M), 20.3 (H);  **Women:** 14.9 (L), 17.8 (M), 19.9 (H) |
|  |  |  |  | Between 50-60 | **Men:** 7.8 (L), 8.7(M), 11.0 (H);  **Women:** 8.1 (L), 9.6 (M), 11.1 (H) |
|  |  |  |  | Between 55-60 | **Men:** 4.3 (L), 4.7 (M), 6.5 (H);  **Women:** 4.6 (L), 5.5 (M), 6.6 (H) |
| Krueger & Slesnick 2014; U.S, | Non-institutional population | Education:  eight groups  (1) 0-12 years of education but no high school diploma or GED,  (2) GED but no high school diploma,  (3) high school diploma  (4) college but no degree,  (5) associate degree but no bachelor’s degree,  (6) bachelor’s degree but no master’s or higher degree,  (7) graduated degree (master or professional degree or PhD | Economic activity expectancy | Between 25-70 | Initially active at labour force  **Men:** 29.4 (1), 30.9 (2), 32.9 (3), 34.5 (4), 35.5 (5), 38.3 (6), 40.2 (7);  **Women:** 19.4 (1), 23.8 (2), 27.3 (3), 29.2 (4), 33.2 (5), 32.7 (6), 35.8 (7);  Initially active at non-work  **Men:** 27.9 (1), 29.5 (2), 31.5 (3), 33.3 (4), 34.4 (5), 37.5 (6), 38.5 (7);  **Women:** 17.6 (1), 22.1 (2), 23.5 (3), 27.1 (4), 31.0 (5), 30.3 (6), 33.1 (7) |
|  |  |  |  | Between 30-70 | Initially active at labour force  **Men:** 25.8 (1), 26.9 (2), 28.8 (3), 30.5 (4), 31.1 (5), 33.9 (6), 35.7 (7);  **Women:** 17.4 (1), 21.1 (2), 24.2 (3), 26.0 (4), 29.2 (5), 28.9 (6), 31.7 (7);  Initially active at non-work  **Men:** 24.2 (1), 25.6 (2), 27.4 (3), 28.9 (4), 29.9 (5), 33.1 (6), 34.8 (7);  **Women:**15.4 (1), 18.9 (2), 21.1 (3), 23.4 (4), 26.7 (5), 25.6 (6), 28.7 (7) |
|  |  |  |  | Between 40-70 | Initially active at labour force  **Men:** 17.8 (1), 19.1 (2), 20.5 (3), 21.9 (4), 22.2 (5), 24.8 (6), 26.4 (7);  **Women:** 12.9 (1), 14.9 (2), 17.9 (3), 19.6 (4), 21.3 (5), 21.3 (6), 23.3 (7);  Initially active at non-work  **Men:** 15.4 (1), 18.0 (2), 18.5 (3), 20.2 (4), 20.7 (5), 22.9 (6), 25.2 (7);  **Women:** 10.4 (1), 12.3 (2), 14.4 (3), 16.7 (4), 18.4 (5), 17.1 (6), 20.1 (7) |
|  |  |  |  | Between 50-70 | Initially active at labour force  **Men:** 11.0 (1), 11.6 (2), 12.7 (3), 14.0 (4), 13.8 (5), 16.0 (6), 17.5 (7);  **Women:** 8.4 (1), 9.7 (2), 11.7 (3), 12.7 (4), 13.5 (5), 13.7 (6), 14.9 (7);  Initially active at non-work  **Men:** 8.2 (1), 9.1 (2), 10.2 (3), 11.1 (4), 11.4 (5), 13.6 (6), 16.1 (7);  **Women:** 5.3 (1), 6.6 (2), 7.9 (3), 9.4 (4), 10.1 (5), 9.6 (6), 11.6 (7) |
|  |  |  |  | Between 55-70 | Initially active at labour force  **Men:** 8.1 (1), 8.2 (2), 9.4 (3), 10.4 (4), 10.1 (5), 12.1 (6), 13.3 (7);  **Women:** 6.5 (1), 7.6 (2), 8.8 (3), 9.4 (4), 10.0 (5), 10.2 (6), 10.9 (7);  Initially active at non-work  **Men:** 5.7 (1), 6.4 (2), 6.5 (3), 8.1 (4), 7.6 (5), 10.0 (6), 11.8 (7);  **Women:** 3.1 (1), 4.6 (2), 4.8 (3), 5.8 (4), 6.3 (5), 6.0 (6), 7.4 (7) |
| Loichinger & Weber 2016;  11 EU countries | General population | Education:  based on ISCED coding-  at most lower secondary education (ISCED 0–2) (low, L);  upper secondary and post-secondary  non-tertiary education (ISCED 3–4) (mediun, M);  short-cycle tertiary  education or higher (ISCED 5–8) (high, H) | Economic activity expectancy | At age 50 | **Men**  Bulgaria: 7.1 (L), 9.9 (M), 12.6 (H);  Chech Republic: 7.4 (L), 10.7 (M), 14.7 (H);  Denmark: 10.5 (L), 11.2 (M), 13.7 (H);  Estonia: 6.4 (L), 10.7 (M), 14.6 (H);  Finland: 8.9 (L), 10.0 (M), 12.1 (H);  Hungary: 4.4 (L), 7.6 (M), 10.2 (H);  Italy: 8.6 (L), 10.7 (M), 14.3 (H);  Norway: 10.1 (L), 13.0 (M), 15.5 (H);  Poland: 6.7 (L), 8.2 (M), 12.7 (H);  Slovenia: 7.6 (L), 8.6 (M), 12.1 (H);  Sweden: 11.9 (L), 13.1 (M), 15.1 (H);  **Women**  Bulgaria: 5.3 (L), 8.6 (M), 10.7 (H);  Chech Republic: 6.3 (L), 8.5 (M), 12.8 (H);  Denmark: 8.4 (L), 10.1 (M), 11.4 (H);  Estonia: 4.5(L), 11.1 (M), 14.3 (H);  Finland: 8.7 (L), 10.4 (M), 11.9 (H);  Hungary: 4.5 (L), 6.7 (M), 9.3 (H);  Italy: 3.9 (L), 7.7 (M), 9.9 (H);  Norway: 8.6 (L), 11.5 (M), 13.3 (H);  Poland: 4.5 (L), 5.6 (M), 9.7 (H);  Slovenia: 5.9 (L), 6.2 (M), 9.8 (H);  Sweden: 9.8 (L), 11.9 (M), 14.0 (H) |
| Özer 2014;  Turkey | General population | Education:  a degree below high (low, L)  a degree from high school or  above (high, H) | Economic activity expectancy | At age 20 | **Women initially active:** 13.79 (L), 22.13 (H);  **Women** i**nitially inactive:** 11.22 (L), 19.48 (H) |
|  |  |  |  | At age 25 | **Women initially active:** 12.73 (L), 19.92 (H);  **Women initially inactive:** 9.64 (L), 16.85 (H) |
|  |  |  |  | At age 30 | **Women initially active:** 11.43 (L), 17.48 (H);  **Women** i**nitially inactive:** 7.85 (L), 13.95 (H) |
|  |  |  |  | At age 35 | **Women initially active:** 10.01 (L), 14.97 (H);  **Women initially inactive:** 6.00 (L), 10.94 (H) |
|  |  |  |  | At age 40 | **Women initially active;** 8.56 (L), 12.53 (H);  **Women initially inactive;** 4.25 (L), 8.01 (H) |
|  |  |  |  | At age 45 | **Women initially active:** 7.17 (L), 10.26 (H);  **Women initially inactive:** 2.76 (L), 5.38 (H) |
|  |  |  |  | At age 50 | **Women initially active:** 5.87 (L), 8.23 (H);  **Women initially inactive:** 1.63 (L), 3.27 (H) |
|  |  |  |  | At age 55 | **Women initially active:** 4.70 (L), 6.45 (H);  **Women initially inactive:** 0.86 (L), 1.77 (H) |
|  |  |  |  | At age 60 | **Women initially active:** 3.64 (L), 4.92 (H);  **Women initially inactive:** 0.40 (L), 0.83 (H) |
|  |  |  |  | At age 65 | **Women initially active:** 2.72 (L), 3.61 (H);  **Women** **initially inactive:** 0.15 (L), 0.32 (H) |
|  |  |  |  | At age 70 | **Women initially active:** 1.88 (L), 2.37 (H);  **Women initially inactive:** 0.04 (L), 0.08 (H) |
| Dudel & Myrskylä 2017;  U.S. | General population | Education:  less than high school degree (low education, L);  a high school diploma or GED (medium, M);  college or university degree (high, H) | Employment expectancy | At age 50 | **Men:** 8.6 (L), 12.1 (M), 15.4 (H);  **Women:** 6.9 (L), 10.2 (M), 12.4 (H) |
| Stanek & Requena 2019; Spain | General population | Education: low-level education (L); medium-level education (M);  high-level education (H) | Employment expectancy | At age 50 | **Men:** 6.6 (L); 9.3 (M); 12.9 (H)  **Women:** 5.2 (L); 7.1 (M); 10.2 (H) |
| van der Noordt et al. 2019; The Netherlands | General population | Education:  elementary school, lower vocational education or less (low, L),  general intermediate, intermediate  vocational, and general secondary education (moderate, M),  higher vocational education, college, and university (high, H) | Employment expectancy | At age 58 | **2012‒2016:** 5.14 (L), 5.43 (M); 5.94 (H) |
|  |  |  | Employment expectancy without disability | At age 58 | **2012‒2016:** 3.32 (L), 3.75 (M); 4.65 (H) |
| Robroek et al. 2020;  The Netherlands | General population | Education:  pre-primary, primary, and lower secondary (low, L):  upper secondary (medium, M) ; post-secondary (high, H) | Employment expectancy | Between 16-66 | **Men:** 29.2 (L), 34.2 (M), 33.4 (H);  **Women:** 23.0 (L), 31.2 (M), 32.5 (H). |
|  |  |  |  | Between 30-66 | **Men:** 20.9 (L), 26.0 (M), 28.2 (H);  **Women:** 16.9 (L), 23.7 (M), 26.8 (H). |
|  |  |  |  | Between 50-66 | **Men**: 8.4 (L), 9.8.0 (M), 10.9 (H);  **Women**: 7.0 (L), 9.1 (M), 10.4 (H). |
| Dudel et al. 2018; Spain | General population | Occupational class:  (highest occupational status ever obtained): ***Year 2012/2013***  skilled non-manual (S n-m), skilled manual (S m), and unskilled non-manual (U n-m), unskilled manual (U m) | Employment expectancy | At age 15 | **Men:** 38.43 (S n-m), 27.01 (S m), 34.33 (U n-m), 27.44 (U m)  **Women:** 35.59 (S n-m), 23.11 (S m), 29.51 (U n-m), 22.16 (U m) |
|  |  |  |  | At age 20 | **Men:** 36.40 (S n-m), 25.93 (S m), 32.91 (U n-m), 26.29 (U m)  **Women:** 33.83 (S n-m), 22.20 (S m), 28.31 (U n-m), 21.14 (U m) |
|  |  |  |  | At age 30 | **Men:** 27.65 (S n-m), 19.67 (S m), 25.45 (U n-m), 20.13 (U m)  **Women:** 25.58 (S n-m), 16.34 (S m), 21.41 (U n-m), 15.33 (U m) |
|  |  |  |  | At age 40 | **Men:** 18.56 (S n-m), 12.37 (S m), 16.82 (U n-m), 12.88 (U m)  **Women:** 17.13 (S n-m), 10.16 (S m), 13.99 (U n-m), 9.39 (U m) |
|  |  |  |  | At age 50 | **Men:** 10.17 (S n-m), 5.95 (S m), 8.68 (U n-m), 6.25 (U m)  **Women:** 9.37 (S n-m), 4.90 (S m), 7.16 (U n-m), 4.21 (U m) |
|  |  |  |  | At age 60 | **Men:** 2.96 (S n-m), 1.25 (S m), 1.98 (U n-m), 1.33 (U m)  **Women:** 2.80 (S n-m), 1.23 (S m), 1.82 (U n-m), 0.74 (U m) |
| Leinonen et al. 2018; Finland | General population | Social class: upper non-manual employees (u n-m), lower non-manual (l n-m), manual workers (m w),  entrepreneurs (e), and others or unknown | Employment expectancy | At age 50 | **Men:** 11.63 (u n-m), 9.54 (l n-m), 7.98 (m w), 10.75 (e)  **Women:** 11.74 (u n-m), 11.07 (l n-m), 8.11 (m w), 9.90 (e). |
| Lorenti et al. 2019; Italy | General population | Occupational class:  managers (m), non-manual (n-m w) and manual workers (m w). ***Year 2012/2013*** | Employment expectancy | At age 15 | **Men:** 36 (m), 31 (n-m w), 26 (m w)  **Women:** 31 (m), 26 (n-m w), 21 (m w) |
| Schram et al. 2021;  Finland | Employed | Occupational class:  upper non-manual employees (u n-m), lower non-manual employees (l n-m), manual workers (mw), self-employed (se). | Productive work expectancy | Between 50–63 | **Men:** 9.5 (mw), 10.08 (l n-m), 10.50 (u n-m), 11.08 (se);  **Women:** 9.51 (mw), 10.12 (l n-m), 10.53 (u n-m), 11.05 (se) |
| Parker et al. 2020;  UK | General population | Education:  less than secondary (low),  secondary (medium),  tertiary (high),  other | Employment expectancy | At age 50 | **Both genders:** 9.38 (L), 22.15 (M), 13.24 (H);  11.96 (n-m), 10.58 (m), 14.42 (s-e) |
|  |  |  | Employment expectancy in good health | At age 50 | **Both genders:** 7.68 (L), 9.54 (M), 11.27 (H);  10.32 (n-m); 8.72 (m); 11.76 (s-e) |
| Lynch et al. 2022;  UK | General population | Occupational class:  non-manual (n-m),  manual (m),  self-employed (s-e) | Employment expectancy | At age 50 | **Both genders:** 11.85 (n-m), 10.35 (m), 13.76 (s-e) |
|  |  |  | Employment expectancy in good health | At age 50 | **Both genders:** 10.06 (n-m); 8.59 (m); 10.98 (s-e) |
|  |  |  |  | At age 55 | **Both genders:** 6.51 (n-m); 5.15 (m); 7.92 (s-e) |
|  |  |  |  | At age 60 | **Both genders:** 3.41 (n-m); 2.77 (m); 4.61 (s-e) |
|  |  |  |  | At age 65 | **Both genders:** 1.16 (n-m); 0.92 (m); 2.14 (s-e) |
|  |  |  |  | At age 70 | **Both genders:** 0.30 (n-m); 0.30 (m); 0.94 (s-e) |
|  |  |  |  | At age 75 | **Both genders:** 0.09 (n-m); 0.10 (m); 0.35 (s-e) |
| Tetzlaff et al. 2022;  Germany | The statutory health insurance receivers | Education:  graduation after 8–11 years of schooling (low, L)  graduation after 12–13 years of schooling (high, H) | Economic activity expectancy | At age 18 | **Men:** 40.06 (L), 37.75 (H);  **Women:** 31.43 (L), 35.46 (H) |
|  |  |  |  | At age 50 | **Men:** 12.18 (L), 13.54 (H);  **Women:** 9.55 (L), 12.62 (H) |
| Schram et al. 2022;  The Netherlands | General population | Education:  primary school, lower and intermediate secondary school, or lower vocational training (low, L),  higher secondary school, or intermediate vocational  training (medium, M),  higher vocational education, or  university education (high, H) | Employment expectancy | Between age 50-66 | **Men:** 12.76 (L), 13.00 (M), 13.20 (H)  **Women:** 12.31 (L), 12.58 (M),12.80 (H) |

Table S4. Results from the included studies on socioeconomic differences in working years lost.

| Author, year, country | Study sample | Operationalization of determinant | Working years lost (WYL) by reason | Age | Results |
| --- | --- | --- | --- | --- | --- |
| Hayward & Grady 1990;  U.S. | In the labour force | Education:  < 12 years (low, L);  13-15 years (medium, M);  > 16 years (high, H). | Retirement | Between 55-70 | **Men:** 11.6 (L), 10.9 (M), 11.2 (H) |
|  |  |  | Disability | Between 55-70 | **Men:** 0.9 (L), 0.8 (M), 0.6 (H). |
| Hayward & Lichter 1998;  U.S | In the labour force | Education:  less than high school (< 12 years, low, L);  more than high school (>16 years, high, H). | Retirement | Between 45-75 | With reentry  **Men:** 8.8 (L), 11.2 (H);  Without reentry  **Men:** 11.3 (L), 14.7 (H) |
|  |  |  |  | Between 55-75 | With reentry  **Men:** 10.0 (L), 11.7 (H);  Without reentry  **Men:** 12.7 (L), 15.1 (H) |
|  |  |  | Disability | Between 45-75 | With reentry  **Men:** 1.1 (L), 0.3 (H);  With reentry  **Men:** 1.2 (L), 0.7 (H); |
|  |  |  |  | Between 55-75 | With reentry  **Men:** 0.8 (L), 0.3 (H);  With reentry  **Men:** 0.8 (L), 0.5 (H) |
| Nurminen 2012;  Finland | General population | Education:  primary or lower secondary education (ISCED levels 1–2), (low, L);  secondary education (ISCED  levels 3–4), (medium, M); tertiary (ISCED  levels 5–6), (high, H) | Unemployment | Between 25-65 | **Men:** 3.3 (L), 2.2 (M), 1.7 (H);  **Women:** 3.4 (L), 2.1 (M), 1.4 (H) |
|  |  |  |  | Between 30-65 | **Men:** 2,7 (L), 1.8 (M), 1.3 (H);  **Women:** 2.8 (L), 1.7 (M), 1.2 (H) |
|  |  |  |  | Between 40-65 | **Men:** 1.6 (L), 1.3 (M), 0.9 (H);  **Women:** 1.6 (L), 1.1 (M), 0.8 (H) |
|  |  |  |  | Between 50-65 | **Men:** 0.8 (L), 0.7 (M), 0.6 (H):  **Women:** 0.7 (L), 0.6 (M), 0.4 (H) |
|  |  |  |  | Between 55-65 | **Men:** 0.5 (L), 0.4 (M), 0.3 (H):  **Women:** 0.4 (L), 0.4 (M), 0.3 (H) |
|  |  |  | Being economically inactive | Between 25-65 | **Men:** 9.5 (L), 7.3 (M), 3.7 (H);  **Women:**12.9 (L), 8.4 (M), 5.9 (H) |
|  |  |  |  | Between 30-65 | **Men:** 8.5 (L), 6.6 (M), 3.5 (H);  **Women:** 10.8 (L), 7.3 (M), 5.1 (H) |
|  |  |  |  | Between 40-65 | **Men**: 7.2 (L), 5.9 (M), 3.3 (H);  **Women:** 8.0 (L), 5.6 (M), 3.9 (H) |
|  |  |  |  | Between 50-65 | **Men**: 5.9 (L), 5.1 (M), 3.0 (H);  **Women**: 5.7 (L), 4.3 (M), 3.0 (H) |
|  |  |  |  | Between 55-65 | **Men:** 4.7 (L), 4.3 (M), 2.7 (H);  **Women**: 4.5 (L), 3.6 (M), 2.6 (H) |
| Dudel & Myrskylä 2017;  U.S | General population | Education:  less than high school degree (low education, L);  a high school diploma or GED (medium, M);  college or university degree (high, H) | Retirement | At age 50 | **Men:** 11.2 (L); 13.9 (M); 14.3 (H);  **Women:** 15.1 (L). 17.5 (M). 19.1 (H) |
|  |  |  | Outside of labour force |  | **Men:** 5.7 (L); 3.6 (M); 2.0 (H);  **Women:** 8.6 (L). 4.9 (M). 2.9 (H) |
| Stanek & Requena 2019; Spain | General population | Education: low-level education (L); medium-level education (M);  high-level education (H) | Unemployment | At age 50 | **Men:** 0.8 (H); 1.8 (M): 2.9 (L)  **Women:** 0.6 (H); 1.3 (M); 2.0 (L) |
|  |  |  | Retirement | At age 50 | **Men:** 19.3 (H); 19.32 (M): 17.2 (L)  **Women**:19.4 (H); 17.4 (M); 17.2 (L) |
|  |  |  | Economically inactive | At age 50 | **Men:** 1.96 (H); 2.4 (M): 3.6 (L);  **Women:** 1.5 (H); 2.2 (M); 3.2 (L) |
| Robroek et al. 2020;  The Netherlands | General population | Education:  pre-primary, primary, and lower secondary (low, L):  upper secondary (medium, M);  post-secondary (high, H). | Unemployment | Between 16-66 | **Men:** 7.7 (L), 3.9 (M), 2.0 (H);  **Women:** 9.3 (L), 4.0 (M), 1.9 (H) |
|  |  |  |  | Between 30-66 | **Men:** 6.3 (L), 3.4 (M), 1.9 (H);  **Women:**7.0 (L), 3.3 (M), 1.8 (H) |
|  |  |  |  | Between 50-66 | **Men**: 2.8 (L), 1.9 (M), 0.3 (H);  **Women**: 2.7 (L), 1.6 (M), 1.1 (H) |
|  |  |  | Disability benefits | Between 16-66 | **Men:** 3.6 (L), 1.9 (M), 0.8 (H);  **Women**: 3.5 (L), 2.3 (M), 1.4 (H) |
|  |  |  |  | Between 30-66 | **Men:** 3.4 (L), 1.8 (M), 0.8 (H);  **Women**: 3.0 (L), 2.0 (M), 1.4 (H) |
|  |  |  |  | Between 50-66 | **Men**: 1.8 (L), 1.1 (M), 0.6 (H);  **Women**: 1.2 (L), 1.0 (M), 0.8 (H) |
|  |  |  | Earlier retirement | Between 16-66 | **Men**: 2.0 (L), 2.3 (M), 2.4 (H);  **Women**: 2.0 (L), 2.1 (M), 2.2 (H) |
|  |  |  |  | Between 30-66 | **Men**: 2.0 (L), 2.3 (M), 2.4 (H);  **Women**: 2.1 (L), 2.1 (M), 2.3 (H) |
|  |  |  |  | Between 50-66 | **Men**: 2.0 (L), 2.3 (M), 2.5 (H);  **Women**: 2.0 (L), 2.1 (M), 2.3 (H) |
|  |  |  | Economically inactive | Between 16-66 | **Men**: 4.7 (L), 5.7 (M), 9.0 (H);  **Women**: 10.4 (L), 9.0 (M), 10.3 (H) |
|  |  |  |  | Between 30-66 | Men: 1.6 (L), 1.2 (M), 1.1 (H);  Women: 6.1 (L), 3.9 (M), 2.6 (H) |
|  |  |  |  | Between 50-66 | Men: 0.4 (L), 0.4 (M), 0.4 (H);  Women: 2.7 (L), 1.9 (M), 1.2 (H) |
| Dudel et al. 2018;  Spain | General population | Occupational class:  (highest occupational status ever obtained):  skilled manual (S m), skilled non-manual, (S n-m) unskilled (U m)  manual, and unskilled non-manual (U n-m) | Unemployment | At age 15 | **Men:** 1.8 (S n-m), 4.1 (S m), 3.5 (U n-m), 7.3 (U m);  **Women:** 2.0 (S n-m), 3.5 (S m), 3.9 (U n-m), 5.6 (U m) |
|  |  |  |  | At age 20 | **Men:** 1.8 (S n-m), 4.0 (S m), 3.5 (U n-m), 7.2 (U m);  **Women:** 1.9 (S n-m), 3.5 (S m), 3.8 (U n-m), 5.6 (U m) |
|  |  |  |  | At age 30 | **Men:** 1.4 (S n-m), 3.4 (S m), 2.9 (U n-m), 6.2 (U m);  **Women:** 1.3 (S n-m), 2.9 (S m), 3.1 (U n-m), 4.4 (U m) |
|  |  |  |  | At age 40 | **Men:** 1.0 (S n-m), 2.5 (S m), 2.2 (U n-m), 4.5 (U m);  **Women:** 0.8 (S n-m), 2.0 (S m), 2.2 (U n-m), 2.8 (U m) |
|  |  |  |  | At age 50 | **Men:** 0.6 (S n-m), 1.5 (S m), 1.4 (U n-m), 2.8 (U m);  **Women:** 0.4 (S n-m), 1.2 (S m), 1.3 (U n-m), 1.5 (U m) |
|  |  |  |  | At age 60 | **Men:** 0.2 (S n-m), 0.4 (S m), 0.4 (U n-m), 0.7 (U m);  **Women:** 0.1 (S n-m), 0.3 (S m), 0.4 (U n-m), 0.6 (U m) |
|  |  |  | Inactive | At age 15 | **Men:** 8.3 (S n-m), 16.8 (S m), 9.8 (U n-m), 13.0 (U m);  **Women:** 11.6 (S n-m), 23.0 (S m), 15.7 (U n-m), 20.0 (U m) |
|  |  |  |  | At age 20 | **Men:** 5.4 (S n-m), 12.9 (S m), 6.3 (U n-m), 9.2 (U m);  **Women:** 8.4 (S n-m), 19.0 (S m), 12.0 (U n-m), 18.1 (U m) |
|  |  |  |  | At age 30 | **Men:** 4.5 (S n-m), 9.9 (S m), 4.3 (U n-m), 6.5 (U m);  **Women:** 7.3 (S n-m), 15.4 (S m), 9.6 (U n-m), 15.1 (U m) |
|  |  |  |  | At age 40 | **Men:** 4.1 (S n-m), 8.2 (S m), 3.7 (U n-m), 5.5 (U m);  **Women:** 6.3 (S n-m), 12.6 (S m), 8.0 (U n-m), 12.7 (U m) |
|  |  |  |  | At age 50 | **Men:** 3.0 (S n-m), 5.7 (S m), 2.8 (U n-m), 4.1 (U m);  **Women:** 4.6 (S n-m), 8.7 (S m), 5.7 (U n-m), 9.3 (U m) |
|  |  |  |  | At age 60 | **Men:** 1.1 (S n-m), 2.1 (S m), 1.1 (U n-m), 1.8 (U m);  **Women:** 1.8 (S n-m), 3.5 (S m), 2.4 (U n-m), 4.2 (U m) |
| Leinonen et al. 2018;  Finland | General population | Social class: upper non-manual employees (u n-m), lower non-manual (l n-m), manual workers (m w),  entrepreneurs (e), and others or unknown | Unemployment | At age 50 | **Men:** 0.82 (u n-m), 1.23 (l n-m), 1.86 (m w), 0.50 (e);  **Women:** 0.54 (u n-m), 0.83 (l n-m), 1.73 (m w), 0.58 (e) |
|  |  |  | Disability retirement |  | **Men:** 0.72 (u n-m), 1.38 (l n-m), 2.71 (m w), 1.65 (e);  **Women:** 0.71 (u n-m), 1.26 (l n-m), 2.79 (m w), 1.97 (e). |
|  |  |  | Other forms of early retirement |  | **Men:** 0.26 (u n-m), 0.32 (l n-m), 0.41 (m w), 0.33 (e);  **Women:** 0.18 (u n-m), 0.39 (l n-m), 0.57 (m w), 0.71 (e) |
|  |  |  | Statuary retirement |  | **Men**: 19.18 (u n-m), 17.77 (l n-m), 14.58 (m w), 16.43 (e);  **Women**: 21.74 (u n-m), 21.87 (l n-m), 19.45 (m w), 19.57 (e) |
|  |  |  | Outside of the labor force for other reasons |  | **Men**: 0.37 (u n-m), 0.59 (l n-m), 0.44 (m w), 0.34 (e);  **Women**: 0.55 (u n-m), 0.24 (l n-m), 0.70 (m w), 0.70 (e) |
| Schram et al. 2021;  Finland | Employed | Occupational class:  upper non-manual employees (u n-m), lower non-manual employees (l n-m), manual workers (mw), self-employed (se). | Time-restricted work disability | Between 50–63 | **Men:** 0.48 (mw), 0.43 (l n-m), 0.38 (u n-m), 0.29 (se);  **Women:** 0.65 (mw), 0.56 (l n-m), 0.48 (u n-m), 0.33 (se) |
|  |  |  | Unemployment | Between 50–63 | **Men:** 1.01 (mw), 0.64 (l n-m), 0.41 (u n-m), 0.17 (se);  **Women:** 1.03 (mw), 0.61 (l n-m), 0.37 (u n-m), 0.15 (se) |
|  |  |  | Economically inactive | Between 50–63 | **Men**: 0.40 (mw), 0.42 (l n-m), 0.43 (u n-m), 0.41 (se);  **Women**: 0.27 (mw), 0.32 (l n-m), 0.36 (u n-m), 0.42 (se) |
|  |  |  | Disability retirement | Between 50–63 | **Men**: 0.83 (mw), 0.68 (l n-m), 0.55 (u n-m), 0.37 (se);  **Women**: 0.79 (mw), 0.63 (l n-m), 0.50 (u n-m), 0.33 (se) |
|  |  |  | Retirement | Between 50–63 | **Men**: 0.55 (mw), 0.55 (l n-m), 0.54 (u n-m), 0.52 (se);  **Women**: 0.66 (mw), 0.67 (l n-m), 0.66 (u n-m), 0.63 (se) |
| Schram et al. 2022 The Netherlands; | General population | Education:  primary school, lower and intermediate secondary school, or lower vocational training (low, L),  higher secondary school, or intermediate vocational  training (medium, M),  higher vocational education, or  university education (high, H) | Involuntary exit (unemployment, disability benefits) | Between 50-66 | **Men** 1.48 (L), 1.31 (M), 1.15 (H);  **Women** 1.76 (L), 1.53 (M), 1.33 (H) |
|  |  |  | Voluntary exit (economic inactivity, earlier retirement) | Between 50-66 | **Men** 1.04 (L), 1.09 (M), 1.14 (H);  **Women** 1.45 (L), 1.40 (M), 1.49 (H) |
| Özer 2014;  Turkey | General population | Education:  a degree below high (low, L)  a degree from high school or  above (high, H) | Inactive | At age 20 | **Women initially active:**  37.48 (L), 29.13 (H);  **Women initially inactive**  40.04 (L), 31.79 (H) |
|  |  |  |  | At age 25 | **Women Initially active::**  33.64 (L), 26.45 (H);  Initially inactive  **Women:** 36.73 (L), 29.52 (H) |
|  |  |  |  | At age 30 | Initially active  **Women:** 30.06 (L), 24.01 (H);  Initially inactive  **Women:** 33.64 (L), 27.54 (H) |
|  |  |  |  | At age 35 | Initially active  **Women:** 26.62 (L), 21.66 (H);  Initially inactive  **Women:** 30.63 (L), 25.69 (H) |
|  |  |  |  | At age 40 | Initially active  **Women:** 23.23 (L), 19.27 (H);  Initially inactive  **Women:** 27.55 (L), 23.79 (H) |
|  |  |  |  | At age 45 | Initially active  **Women:** 19.85 (L), 16.76 (H);  Initially inactive  **Women:** 24.26 (L), 21.64 (H) |
|  |  |  |  | At age 50 | Initially active  **Women:** 16.45 (L), 14.10 (H);  Initially inactive  **Women:** 20.70 (L), 19.05 (H) |
|  |  |  |  | At age 55 | Initially active  **Women:** 13.04 (L), 11.29 (H);  Initially inactive  **Women:** 16.88 (L), 15.97 (H) |
|  |  |  |  | At age 60 | Initially active  **Women:** 9.64 (L), 8.36 (H);  Initially inactive  **Women:** 12.89 (L), 12.46 (H) |
|  |  |  |  | At age 65 | Initially active  **Women:** 6.23 (L), 5.34 (H);  Initially inactive  **Women:** 8.79 (L), 8.63 (H) |
|  |  |  |  | At age 70 | Initially active  **Women:** 2.75 (L), 2.25 (H);  Initially inactive  **Women:** 4.58 (L), 4.55 (H) |

**Figure S1.** Educational differences in WYL due to unemployment, disability retirement and other type of retirement among men and women.


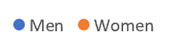


**
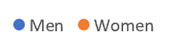
**
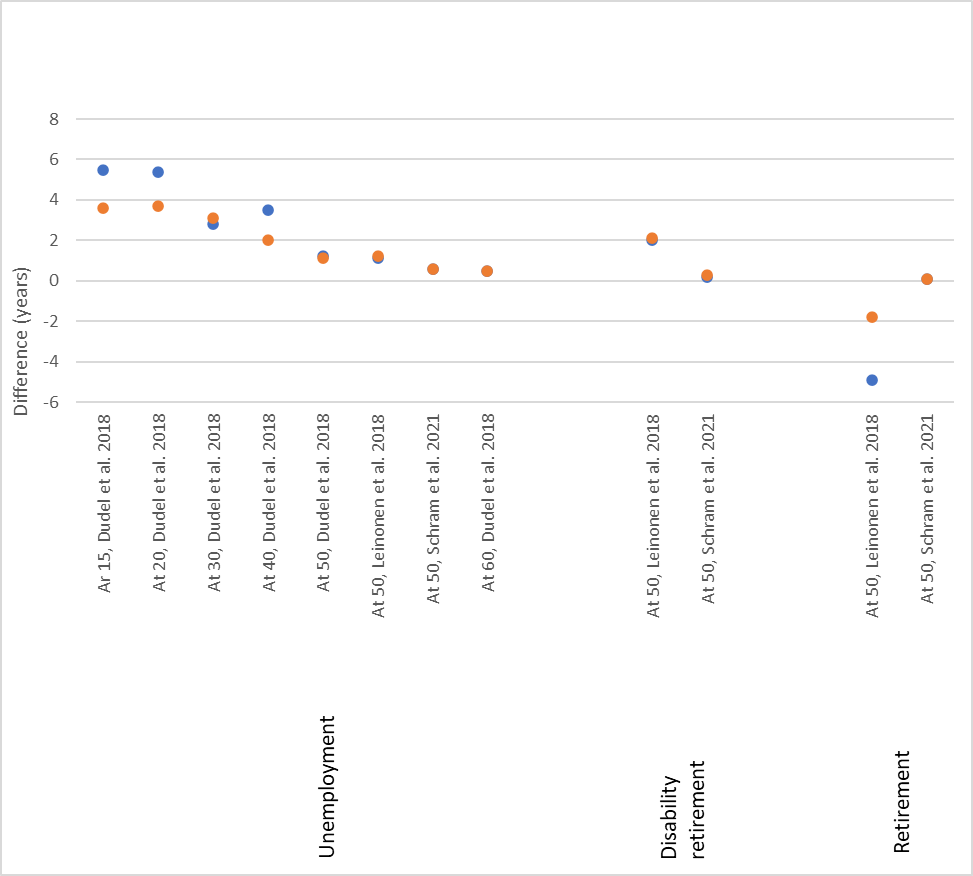
**Figure S2.** Occupational class differences in WYL due to unemployment, disability retirement and other type of retirement among men and women.

**REFERENCES**

Dudel C, Loichinger E, Klüsener S, Sulak H, Myrskylä M. The extension of late working life in Germany: trends, inequalities, and the East-West divide. Rostock: Max Planck Institute for Demographic Research (MPIDR). MPIDR Working Paper 2021;No.WP-2021-018. Available at https://doi.org/10.4054/MPIDR-WP-2021-018 or https://nbn-resolving.org/urn:nbn:de:0168-ssoar-77024-2 (Accessed on 10.07.2023)

Dudel C, López Gómez MA, Benavides FG, Myrskylä M. The Length of Working Life in Spain: Levels, Recent Trends, and the Impact of the Financial Crisis. Eur J Popul. 2018;34:769-91.

Dudel C, Myrskylä M. Working Life Expectancy at Age 50 in the United States and the Impact of the Great Recession. Demography. 2017;54:2101-23.

Hayward MD, Grady WR. Work and retirement among a cohort of older men in the United States, 1966-1983. Demography. 1990;27:337-56.

Hayward MD, Lichter A life cycle model of labour force inequality. Extending Clogg’s life table approach. Soc Meth Res. 1998:26:480-510.

Karlsson M, Mayhew L, Rickayzen B. Individualised life tables. Investigating dynamics of health, work and cohabitation in the UK. Pop Ageing, 2009;1:153–91.

Krueger KV, Slesnick F. Total worklife expectancy. J Forensic Economics. 2014;25:51-70.

Leinonen T, Martikainen P, Myrskylä M. Working Life and Retirement Expectancies at Age 50 by Social Class: Period and Cohort Trends and Projections for Finland. J Gerontol B Psychol Sci Soc Sci. 2018;73:302-13.

Loichinger E, Weber D. Trends in Working Life Expectancy in Europe. J Aging Health. 2016;28:1194-213.

Lorenti A, Dudel C, Myrskyla M. The Legacy of the Great Recession in Italy: A Wider Geographical, Gender, and Generational Gap in Working Life Expectancy. Soc Indic Res. 2019;142: 283-303.

Lorenti A, Dudel C Hale JM, Myrskylä M. Working and disability expectancies at older ages: The role of childhood circumstances and education. Soc Sci Res 2020; 91:102447.

Lynch M, Bucknall M, Jagger C, Wilkie R. Healthy working life expectancy at age 50 for people with and without osteoarthritis in local and national English populations. Sci Rep 2022; 12(1):2408.

Millimet DL, Nieswiadomy M, Ryu H, Slottje D. Estimating worklife expectancy: an econometric approach. J Econometrics. 2003;113: 83-113.

Millimet D L, Nieswiadomy M, Slottje DJ. Detailed estimation of worklife expectancy for the measurement of human capital: accounting for marriage and children. J Econom Surveys. 2010;24:339-61.

Nexø MA, Pedersen J, Cleal B, Andersen I, Bjørner JB. Working life expectancies among individuals with type 1 and type 2 diabetes over a 30-year period. Scand J Work Environ Health. 2021; 47:540-9.

Nurminen M. Working-life expectancy in Finland: trends and differentials 2000–2015. A multistate regression modeling approach. Helsinki: Finnish Centre for Pensions; 2012. Finnish Centre for Pensions report no 3. Available at: https://www.julkari.fi/bitstream/handle/10024/129432/WorkinglifeexpectancyinFinlandtrendsanddifferentials20002015Amultistateregressionmodelingapproach.pdf?sequence=1&isAllowed=y (Accessed on 10.07.2023)

Parker M, Bucknall M, Jagger C, Wilkie R. Population-based estimates of healthy working life expectancy in England at age 50 years: analysis of data from the English Longitudinal Study of Ageing. Lancet Public Health, 2020; 5:e395-403.

Robroek SJ, Nieboer D, Järvholm B, Burdorf A. Educational differences in duration of working life and loss of paid employment: working life expectancy in The Netherlands. Scand J Work Environ Health. 2020;46:77-84.

Schram JL, Solovieva S, Leinonen T, Viikari-Juntura E, Burdorf A, Robroek SJ. The influence of occupational class and physical workload on working life expectancy among older employees. Scand J Work Environ Health. 2021;47:5-14.

Schram JLD, Schuring M, Oude Hengel KM, Burdorf A, Robroek SJW. The influence of chronic diseases and poor working conditions in working life expectancy across educational levels among older employees in the Netherlands. Scand J Work Environ Health. 2022;48:391-8.

Skoog GR, Ciecka JE, and Krueger KV. The Markov Process Model of Labor Force Activity: Extended Tables of Central Tendency, Shape, Percentile Points, and Bootstrap Standard Errors. J Forensic Economics. 2011;22:165-229.

Stanek M, Requena M. Expected Lifetime in Different Employment Statuses: Evidence From the Economic Boom-and-Bust Cycle in Spain. Res Aging. 2019;41:286-309.

Tetzlaff J, Luy M, Epping J, Geyer S, Beller J, Stahmeyer JT, et al. Estimating trends in working life expectancy based on health insurance data from Germany - Challenges and advantages. SSM Popul Health. 2022;19:101215.

van der Noordt M, van der Pas S, van Tilburg TG, van den Hout A, Deeg DJ. Changes in working life expectancy with disability in the Netherlands, 1992-2016. Scand J Work Environ Health. 2019;45:73-81.

Weber D, Loichinger E. Live longer, retire later? Developments of healthy life expectancies and working life expectancies between age 50–59 and age 60–69 in Europe. Eur J Ageing. 2020;19:75-93.

Özer MN. The Effects of Education and Marital Status on Female Working Life Expectancy in Turkey. An application of multistate life table for 2009-2010. Ankara: Thesis Center: 2014. Master thesis no 356184. Available at: <https://tez.yok.gov.tr/UlusalTezMerkezi/tezSorguSonucYeni.jsp> (Accessed on 10.07.2023)
